# Healthcare Professionals' Perceptions of Artificial Intelligence in Healthcare—A Systematic Review of Qualitative Studies

**DOI:** 10.1111/jan.70500

**Published:** 2026-01-26

**Authors:** Huotari Sini, Mikkonen Kristina, Jarva Erika, Suonnansalo Petra, Kaikkonen Assi, Lee Jay Jung Jae, Oikarinen Anne

**Affiliations:** ^1^ Research Unit of Health Sciences and Technology, Faculty of Medicine University of Oulu Oulu Finland; ^2^ Medical Research Center Oulu Oulu University Hospital and University of Oulu Oulu Finland; ^3^ School of Nursing The University of Hong Kong Hong Kong China; ^4^ The Finnish Centre for Evidence‐Based Health Care: A Joanna Briggs Institute Centre of Excellence Group Helsinki Finland

**Keywords:** artificial intelligence, healthcare professionals, meta‐aggregation, perceptions, qualitative literature review, systematic review

## Abstract

**Aim:**

To identify the experiences and perceptions of healthcare professionals on artificial intelligence in healthcare.

**Design:**

Systematic literature review of qualitative studies and meta‐aggregation.

**Data Sources:**

CINAHL, PubMed, Scopus, Medic and ProQuest were systematically searched on December 9, 2024.

**Results:**

Twenty‐six studies were included in the review, of which 25 were analysed using meta‐aggregation, and the results of one study were reported narratively. A total of 185 findings were identified from the included studies that addressed the research question. These findings were aggregated into 33 categories and then into five synthesised findings as follows: (1) Perceived benefits of AI in healthcare; (2) Perceived impact of AI on professional roles and workforce dynamics; (3) Perceived impacts of AI in communication and interaction; (4) Perceived challenges of AI related to technical, financial and systemic factors; (5) Perceived ethical, cultural and regulatory considerations regarding the use of AI.

**Conclusion:**

While AI holds significant potential to enhance efficiency and improve patient outcomes, it is essential to address the concerns raised by healthcare professionals regarding workforce dynamics, communication and ethical considerations.

**Implications for the Profession:**

The results can inform and support the implementation of AI in healthcare and the development of AI‐related education and training to meet the demands of future healthcare work.

**Reporting Method:**

The review was conducted and reported in accordance with the PRISMA guidelines.

**Patient or Public Contribution:**

None.

**Trial Registration:**

PROSPERO (CRD1073200)

## 1 Introduction

Artificial intelligence (AI) is recognised as a key element in the digital transformation of society, offering economic and societal benefits across the entire spectrum of industries (European Union 2024), and its potential in the context of healthcare is also evident. According to the World Health Organization's (WHO) State of the World's Nursing Report 2025, the global nursing workforce faced an estimated shortage of 5.8 million nurses in 2023 (WHO 2025). It is projected that by 2030, the global shortage of healthcare workers will reach 10 million (Boniol et al. 2022). Additionally, almost half of healthcare professionals' working time is currently spent interacting with various electronic health record systems (Budd 2023; Toscano et al. 2020). Therefore, it is crucial to develop digital solutions that enable healthcare professionals to have more time on direct patient care and meaningful interaction with patients. In recent years, the development of AI technologies has progressed rapidly, and their adoption in healthcare has become more feasible (Chhibber et al. 2025). Applications have expanded from diagnostic and decision support tools to administrative workflows, making the integration of AI into everyday clinical practice increasingly common.

AI is a multifaceted concept that encompasses a wide range of methods, applications, technologies and research directions (Boucher 2020). According to the European Union, AI systems refer to machine‐based systems designed to operate with varying levels of autonomy. The AI system infers from the input it receives how to generate outputs such as content, recommendations, predictions or decisions that can influence physical or virtual environments (European Union 2024). Machine learning (ML) can be considered as a subfield of AI where computers learn patterns and form associations based on data (Rudner and Toner 2021). Other areas of the practical application of AI include: (1) computer vision, which relates to the automation of image recognition, analysis and interpretation of visual information, (2) natural language processing (NLP) which considers the automation of reading, analysis and generation of human language, (3) speech recognition concerned with the detection, analysis and interpretation of spoken human language and (4) robotics, which brings together different types of AI with the addition of manipulating physical objects (Sheikh et al. 2023). Due to the broad nature of the definition of AI and the lack of precise boundaries, this study does not exclude any area or type of AI.

According to previous studies, AI and its applications are seen to offer several opportunities in healthcare. It can support the automation of repetitive tasks, improve medication accuracy and facilitate clinical decision‐making among other applications (Wubineh et al. 2023). The use of computer vision has improved the accuracy and reduced the time spent on diagnostics in radiology and pathology, streamlining and improving workflow efficiencies (Jeong et al. 2025). Speech recognition and NLP techniques have been used in automating clinical documentation to reduce administrative burden and clinician burnout with promising results (Olson et al. 2025; van Buchem et al. 2021). Care robots have been reported to free nursing time for direct care, especially in logistics‐related and routine tasks (Adeyemo et al. 2025). The implementation of AI systems also raises several challenges, such as ethical concerns and issues related to data protection, unreliability of technology (Wubineh et al. 2023), patient safety (Adeyemo et al. 2025) and data quality (Silcox et al. 2024). Moreover, there is still limited evidence about the effects of AI on the clinical roles, human expertise and skills (upskilling vs. deskilling), especially from the perspective of healthcare professionals outside medicine (Miller and Mutha 2025). Addressing these challenges is essential for the successful and meaningful implementation of AI, especially considering healthcare professionals' direct and indirect exposure to AI in their daily work. Healthcare professionals' perceptions are crucial in the development of AI‐driven solutions in healthcare, as they offer valuable insights into practical experiences, patient safety and educational needs related to AI. Moreover, the perceptions and attitudes also influence how effectively AI can be integrated into healthcare in the future. Therefore, it is essential to explore and understand the views of healthcare professionals on AI.

A preliminary search of the literature (PROSPERO, CINAHL, PubMed) revealed that several previously published systematic reviews have examined healthcare professionals' perceptions of AI; however, most are limited in scope. Reviews have been conducted on the perceptions and attitudes of radiologists and medical students (Santomartino and Yi 2022), dentists and dental students (Dashti et al. 2024), as well as healthcare students more broadly (Mousavi Baigi et al. 2023). In addition, reviews including the perspectives from multiple stakeholder groups on AI provide useful insights but fail to exclusively address healthcare professionals' viewpoints (Beets et al. 2023; Chew and Achananuparp 2022; Hogg et al. 2023; Vo et al. 2023). In systematic reviews focusing exclusively on healthcare professionals' perspectives, the scope of AI has been limited to a specific type of AI (Ayorinde et al. 2024) or a particular clinical setting, such as mental health services (Rogan, Bucci, and Firth 2024). Synthesising the qualitative studies addressing healthcare professionals' perceptions towards AI gives a valuable contribution in understanding the underlying expectations, concerns and motivations in AI system adoption and use to guide workforce adjustment to the rapidly changing healthcare environment. To address this gap, this systematic review seeks to comprehensively synthesise current qualitative evidence on healthcare professionals' perceptions of AI in healthcare across diverse roles, technologies and settings.

## 2 Aim

This review aims to identify the perceptions of healthcare professionals on AI in healthcare. The research question of the study is: What kind of experiences and perceptions do healthcare professionals have on AI in healthcare?

## 3 Methods

### 3.1 Study Design

This systematic review was conducted in accordance with the published PROSPERO protocol (CRD1073200) and adhered to the Joanna Briggs Institute (JBI) guidelines for systematic reviews of qualitative evidence (Porritt et al. 2024). The Preferred Reporting Items for Systematic reviews and Meta‐Analyses (PRISMA) statement was used when reporting the process of selecting the articles (Page et al. 2021).

### 3.2 Search Strategy

The search strategy was created using the PICo (population, phenomena of interest, context) strategy for systematic reviews of qualitative evidence (Porritt et al. 2024). Various search terms and combinations of terms were tested, and the final search was conducted with the assistance of an information specialist. The final search was conducted on December 9, 2024, using five databases: CINAHL, PubMed, Scopus, ProQuest and the Finnish database Medic, containing health sciences publications published in Finnish national scientific journals. These databases were selected to attain a broad scope of relevant studies in multidisciplinary databases (Scopus and ProQuest) as well as discipline‐specific databases (PubMed, CINAHL). The search strategies by database are presented in Table 1.

**TABLE 1:** Search strategies by database.

Search terms	Results retrieved
**CINAHL**	
((“Health Personnel”[MeSH Terms] OR “health care worker*”[Text Word] OR “healthcare worker*”[All Fields] OR “health worker*”[Text Word] OR “healthcare professional*”[Text Word] OR “health care professional*”[Text Word] OR “health professional*”[Text Word] OR “health care personnel*”[Text Word] OR “healthcare personnel*”[Text Word] OR “health personnel*”[Text Word] OR “physician*”[Text Word] OR “doctor*”[Text Word] OR “clinician*”[Text Word] OR “general practitioner*”[Text Word] OR “nurse*”[Text Word] OR “midwife*”[Text Word] OR “paramedic*”[Text Word] OR “dentist*”[Text Word] OR “dental hygienist*”[Text Word] OR “pharmacist*”[Text Word] OR “physiotherapist*”[Text Word] OR “dietician*”[Text Word] OR “audiologist*”[Text Word] OR “optometrist*”[Text Word] OR “occupational therapist*”[Text Word]) AND (“english”[Language] OR “finnish”[Language]) AND ((“Artificial Intelligence”[MeSH Terms] OR “artificial intelligenc*”[Text Word] OR “AI”[Text Word] OR “machine learning”[Text Word] OR “machine intelligenc*”[Text Word] OR “natural language processing”[Text Word] OR “NLP”[Text Word] OR “predictive analysis”[Text Word] OR “deep learning”[Text Word] OR “generative artificial intelligenc*”[Text Word] OR “generative AI”[Text Word] OR “ChatGPT”[Text Word] OR “Chat GPT”[Text Word] OR “chatbot*”[Text Word] OR “computational intelligenc*”[Text Word] OR “cognitive computing”[Text Word] OR “robotic*”[Text Word] OR “expert system*”[Text Word] OR “neural network*”[Text Word] OR “data scienc*”[Text Word] OR “autonomous system*”[Text Word] OR “cybernetic*”[Text Word] OR “humanoid intelligenc*”[Text Word] OR “hybrid intelligenc*”[Text Word]) AND (“english”[Language] OR “finnish”[Language])) AND ((“Attitude of Health Personnel”[MeSH Terms] OR “perception*”[Text Word] OR “experience*”[Text Word] OR “attitude*”[Text Word] OR “view*”[Text Word] OR “perspective*”[Text Word] OR “feel*”[Text Word] OR “insight*”[Text Word] OR “belief*”[Text Word] OR “opinion*”[Text Word] OR “expectation*”[Text Word] OR “thought*”[Text Word]) AND (“english”[Language] OR “finnish”[Language])) AND ((“Qualitative Research”[MeSH Terms] OR “Interviews as Topic”[MeSH Terms] OR “qualitative*”[Text Word] OR “ethnol*”[Text Word] OR “ethnog*”[Text Word] OR “ethnonur*”[Text Word] OR “phenomenolog*”[Text Word] OR “phenomenograph*”[Text Word] OR “hermeneutic*”[Text Word] OR “lived experience*”[Text Word] OR “field note*”[Text Word] OR “field record*”[Text Word] OR “field stud*”[Text Word] OR “observati*”[Text Word] OR “participant observ*”[Text Word] OR “grounded theory”[Text Word] OR “constant compar*”[Text Word] OR “focus group*”[Text Word] OR “narrati*”[Text Word] OR “interview*”[Text Word] OR “conversation*”[Text Word] OR “unstructured interview*”[Text Word] OR “semi structured interview*”[Text Word] OR “metasynthes*”[Text Word] OR “meta synthes*”[Text Word] OR “metasummar*”[Text Word] OR “meta summar*”[Text Word] OR “metastud*”[Text Word] OR “meta stud*”[Text Word] OR “meta ethnograph*”[Text Word] OR “metaethnog*”[Text Word] OR “meta narrative*”[Text Word] OR “metanarrat*”[Text Word] OR “meta interpretation*”[Text Word] OR “metainterpret*”[Text Word] OR “qualitative meta analy*”[Text Word] OR “content analy*”[Text Word] OR “thematic analy*”[Text Word] OR “mixed method*”[Text Word] OR “mixed model*”[Text Word] OR “mixed design*”[Text Word] OR “multiple method*”[Text Word] OR “multimethod*”[Text Word] OR “triangulat*”[Text Word]) AND (“english”[Language] OR “finnish”[Language]))) AND (english[Filter] OR finnish[Filter])	#872
**PubMed**	
((“Health Personnel”[MeSH Terms] OR “health care worker*”[Text Word] OR “healthcare worker*”[All Fields] OR “health worker*”[Text Word] OR “healthcare professional*”[Text Word] OR “health care professional*”[Text Word] OR “health professional*”[Text Word] OR “health care personnel*”[Text Word] OR “healthcare personnel*”[Text Word] OR “health personnel*”[Text Word] OR “physician*”[Text Word] OR “doctor*”[Text Word] OR “clinician*”[Text Word] OR “general practitioner*”[Text Word] OR “nurse*”[Text Word] OR “midwife*”[Text Word] OR “paramedic*”[Text Word] OR “dentist*”[Text Word] OR “dental hygienist*”[Text Word] OR “pharmacist*”[Text Word] OR “physiotherapist*”[Text Word] OR “dietician*”[Text Word] OR “audiologist*”[Text Word] OR “optometrist*”[Text Word] OR “occupational therapist*”[Text Word]) AND (“english”[Language] OR “finnish”[Language]) AND ((“Artificial Intelligence”[MeSH Terms] OR “artificial intelligenc*”[Text Word] OR “AI”[Text Word] OR “machine learning”[Text Word] OR “machine intelligenc*”[Text Word] OR “natural language processing”[Text Word] OR “NLP”[Text Word] OR “predictive analysis”[Text Word] OR “deep learning”[Text Word] OR “generative artificial intelligenc*”[Text Word] OR “generative AI”[Text Word] OR “ChatGPT”[Text Word] OR “Chat GPT”[Text Word] OR “chatbot*”[Text Word] OR “computational intelligenc*”[Text Word] OR “cognitive computing”[Text Word] OR “robotic*”[Text Word] OR “expert system*”[Text Word] OR “neural network*”[Text Word] OR “data scienc*”[Text Word] OR “autonomous system*”[Text Word] OR “cybernetic*”[Text Word] OR “humanoid intelligenc*”[Text Word] OR “hybrid intelligenc*”[Text Word]) AND (“english”[Language] OR “finnish”[Language])) AND ((“Attitude of Health Personnel”[MeSH Terms] OR “perception*”[Text Word] OR “experience*”[Text Word] OR “attitude*”[Text Word] OR “view*”[Text Word] OR “perspective*”[Text Word] OR “feel*”[Text Word] OR “insight*”[Text Word] OR “belief*”[Text Word] OR “opinion*”[Text Word] OR “expectation*”[Text Word] OR “thought*”[Text Word]) AND (“english”[Language] OR “finnish”[Language])) AND ((“Qualitative Research”[MeSH Terms] OR “Interviews as Topic”[MeSH Terms] OR “qualitative*”[Text Word] OR “ethnol*”[Text Word] OR “ethnog*”[Text Word] OR “ethnonur*”[Text Word] OR “phenomenolog*”[Text Word] OR “phenomenograph*”[Text Word] OR “hermeneutic*”[Text Word] OR “lived experience*”[Text Word] OR “field note*”[Text Word] OR “field record*”[Text Word] OR “field stud*”[Text Word] OR “observati*”[Text Word] OR “participant observ*”[Text Word] OR “grounded theory”[Text Word] OR “constant compar*”[Text Word] OR “focus group*”[Text Word] OR “narrati*”[Text Word] OR “interview*”[Text Word] OR “conversation*”[Text Word] OR “unstructured interview*”[Text Word] OR “semi structured interview*”[Text Word] OR “metasynthes*”[Text Word] OR “meta synthes*”[Text Word] OR “metasummar*”[Text Word] OR “meta summar*”[Text Word] OR “metastud*”[Text Word] OR “meta stud*”[Text Word] OR “meta ethnograph*”[Text Word] OR “metaethnog*”[Text Word] OR “meta narrative*”[Text Word] OR “metanarrat*”[Text Word] OR “meta interpretation*”[Text Word] OR “metainterpret*”[Text Word] OR “qualitative meta analy*”[Text Word] OR “content analy*”[Text Word] OR “thematic analy*”[Text Word] OR “mixed method*”[Text Word] OR “mixed model*”[Text Word] OR “mixed design*”[Text Word] OR “multiple method*”[Text Word] OR “multimethod*”[Text Word] OR “triangulat*”[Text Word]) AND (“english”[Language] OR “finnish”[Language]))) AND (english[Filter] OR finnish[Filter])	#1966
**Scopus**	
(TITLE‐ABS‐KEY (“health care worker*” OR “healthcare worker*” OR “health worker*” OR “healthcare professional*” OR “health care professional*” OR “health professional*” OR “health care personnel*” OR “healthcare personnel*” OR “health personnel*” OR physician* OR doctor* OR clinician* OR “general practitioner*” OR nurse* OR midwife* OR paramedic* OR dentist* OR “dental hygienist*” OR pharmacist* OR physiotherapist* OR dietician* OR audiologist* OR optometrist* OR “occupational therapist*”) AND TITLE‐ABS‐KEY (“artificial intelligenc*” OR ai OR “machine learning” OR “machine intelligenc*” OR “natural language processing” OR nlp OR “predictive analysis” OR “deep learning” OR “generative artificial intelligenc*” OR “generative AI” OR chatgpt OR “Chat GPT” OR chatbot* OR “computational intelligenc*” OR “cognitive computing” OR robotic* OR “expert system*” OR “neural network*” OR “data scienc*” OR “autonomous system*” OR cybernetic* OR “humanoid intelligenc*” OR “hybrid intelligenc*” OR “artificial health technolog*”) AND TITLE‐ABS‐KEY (perception* OR experience* OR attitude* OR view* OR perspective* OR feel* OR insight* OR belief* OR opinion* OR expectation* OR thought*) AND TITLE‐ABS‐KEY (qualitative* OR ethnol* OR ethnog* OR ethnonur* OR phenomenolog* OR phenomenograph* OR hermeneutic* OR “lived experience*” OR “field note*” OR “field record*” OR “field stud*” OR observati* OR “participant observ*” OR “grounded theory” OR “constant compar*” OR “focus group*” OR narrati* OR interview* OR conversation* OR “unstructured interview*” OR “semi‐structured interview* “OR metasynthes* OR “meta‐synthes*” OR metasummar* OR “meta‐summar*” OR metastud* OR “meta‐stud*” OR “meta‐ethnograph*” OR metaethnog* OR “meta‐narrative*” OR metanarrat* OR “meta‐interpretation*” OR metainterpret* OR “qualitative meta‐analy*” OR “content analy*” OR “thematic analy*” OR “mixed method*” OR “mixed model*” OR “mixed design*” OR “multiple method*” OR multimethod* OR triangulat*))	#3216
**Medic**	
terveydenh* healthcare* ammatti* professional* sairaanhoitaj* lääkär* hoitohenkilökun* hoitaj* AND tekoäly* “artificial intelligence” AI koneoppiminen “machine learning” chatbot* chattibot*	#56
**ProQuest**	
noft(“health care worker*” OR “healthcare worker*” OR “health worker*” OR “healthcare professional*” OR “health care professional*” OR “health professional*” OR “health care personnel*” OR “healthcare personnel*” OR “health personnel*” OR physician* OR doctor* OR clinician* OR “general practitioner*” OR nurse* OR midwife* OR paramedic* OR dentist* OR “dental hygienist*” OR pharmacist* OR physiotherapist* OR dietician* OR audiologist* OR optometrist* OR “occupational therapist*”) AND noft(“artificial intelligenc*” OR AI OR “machine learning” OR “machine intelligenc*” OR “natural language processing” OR NLP OR “predictive analysis” OR “deep learning” OR “generative artificial intelligenc*” OR “generative AI” OR ChatGPT OR “Chat GPT” OR chatbot* OR “computational intelligenc*” OR “cognitive computing” OR robotic* OR “expert system*” OR “neural network*” OR “data scienc*” OR “autonomous system*” OR cybernetic* OR “humanoid intelligenc*” OR “hybrid intelligenc*” OR “artificial health technolog*”) AND noft(perception* OR experience* OR attitude* OR view* OR perspective* OR feel* OR insight* OR belief* OR opinion* OR expectation* OR thought*) AND noft(qualitative* OR ethnol* OR ethnog* OR ethnonur* OR phenomenolog* OR phenomenograph* OR hermeneutic* OR “lived experience*” OR “field note*” OR “field record*” OR “field stud*” OR observati* OR “participant observ*” OR “grounded theory” OR “constant compar*” OR “focus group*” OR narrati* OR interview* OR conversation* OR “unstructured interview*” OR “semi‐structured interview* “OR metasynthes* OR “meta‐synthes*” OR metasummar* OR “meta‐summar*” OR metastud* OR “meta‐stud*” OR “meta‐ethnograph*” OR metaethnog* OR “meta‐narrative*” OR metanarrat* OR “meta‐interpretation*” OR metainterpret* OR “qualitative meta‐analy*” OR “content analy*” OR “thematic analy*” OR “mixed method*” OR “mixed model*” OR “mixed design*” OR “multiple method*” OR multimethod* OR triangulat*)	#1755

### 3.3 Inclusion and Exclusion Criteria

The inclusion criteria were chosen according to the PICo protocol as follows: Population (P) consisted of healthcare professionals. Healthcare professionals were defined according to the WHO's International Classification of Health Workers. According to the classification, health professionals provide preventive, curative, rehabilitative and promotional care based on extensive knowledge of diagnosis and treatment. These professionals include doctors, nurses, midwives, traditional and complementary medicine practitioners, paramedical practitioners, dentists, pharmacists, environmental and occupational health and hygiene professionals, physiotherapists, dietitians, nutritionists, audiologists, speech therapists, optometrists and ophthalmic opticians. In addition, medical imaging and therapeutic equipment technicians (e.g., radiographers) and medical laboratory technicians, who the WHO classifies as health associate professionals, were included in the study (WHO 2019). Studies that examined the perspectives of patients, family members, students or different stakeholders were excluded when the results were not reported separately from healthcare professionals.

Phenomena of interest (I) were healthcare professionals' perceptions of AI. In this study, perceptions are understood to encompass experiences, attitudes, views, perspectives, opinions and expectations regarding AI and its use in clinical practice. The context (Co) considered all healthcare settings, including primary care, specialised care and services provided by the third sector (e.g., non‐governmental health organisations). Digital healthcare environments within these settings were also included.

Further, the review included studies that used qualitative methods including descriptive studies, phenomenology, grounded theory, ethnography, action research and feminist research. Qualitative results from mixed‐method studies were also included. Studies published in English and Finnish were considered with no date limitations.

### 3.4 Search Outcome

**FIGURE 1:**
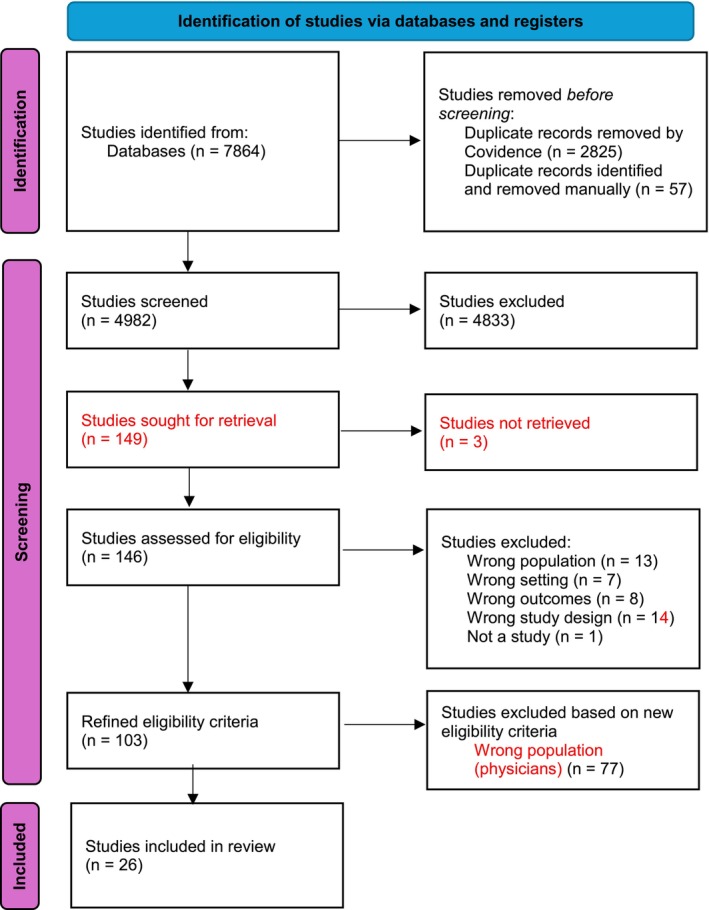
PRISMA flow diagram.

The search identified 7864 studies from databases that were imported into Covidence, a systematic review management tool, for title and abstract screening, full‐text review and data extraction. After removing duplicates (*n* = 2882), a total of 4982 study titles and abstracts were screened by two independent researchers using the established inclusion and exclusion criteria. A total of 146 studies were included for full‐text review as no full‐text was available for three studies. At this point, while the original inclusion criteria covered all healthcare professionals, physicians were excluded after full‐text review when the eligibility criteria were refined. This led to the exclusion of 77 studies. The decision was based on consensus within the review team to ensure a manageable volume of data and, simultaneously, a more focused and coherent synthesis of the experiences and perceptions of multiple healthcare professions. The inclusion of such a large number of studies with only physicians as the major population would have diminished the findings from other healthcare professions. Ultimately, 26 studies were included in the review to represent the experiences and perceptions of healthcare professionals outside medicine. The search process is presented in the PRISMA flow diagram (Figure 1).

### 3.5 Quality Appraisal

The JBI Critical Appraisal Checklist for Qualitative research was used to assess the methodological quality of the studies (Lockwood et al. 2015). Two researchers (SH and KM, AO, EJ, JL, AK, SP) independently evaluated each study. The checklist included 10 questions, each scored ‘yes (= Y)’, ‘no (= N)’ or ‘unclear’. The third researcher (KM, AO, EJ, JL, AK, SP) resolved disagreements that occurred in a total of 19 studies. The methodological quality of studies was not used as a basis for exclusion in this review. Therefore, all appraised studies were retained regardless of quality.

### 3.6 Data Extraction

The data from the chosen studies were extracted based on the details reported in each study by one reviewer and later confirmed by the other authors. The studies were repeatedly read to get fully acquainted with the contents. The extracted details included authors, publication year and study design, methods for data collection and analysis, geographical location, phenomena of interest, participants and main results of the study. Data extraction is presented in Table 2.

**TABLE 2:** Data extraction.

Study	Methods for data collection	Data analysis	Country	Phenomena of interest	Participant characteristics and sample size	Main results
Akudjedu et al. (2023)	An exploratory cross‐sectional study with an online survey designed to obtain both quantitative and qualitative responses	Descriptive statistics and thematic analysis	United Kingdom	Radiography professionals' knowledge, perceptions and expectations of AI technologies	190 radiographers out of a total of 314 radiography professionals	The benefits of AI in radiography related to enhanced workflows and optimised workstreams while the misgivings revolve around de‐skilling and impact on patient‐centred care due to over‐reliance on advanced technology following AI implementation.
Amin et al. (2025)	A mixed‐method study with quantitative survey and qualitative semi‐structured interviews	Descriptive and inferential statistics and thematic analysis	Egypt	The sentiment of nurses towards the utilisation of AI	500 nurses (17 nurses in qualitative study)	Qualitatively, nurses cited obstacles such as lack of familiarity with AI technologies, biases affecting decision‐making, technological challenges, inadequate training and fear of technology replacing human interaction.
Badawy and Shaban (2025)	A phenomenological qualitative study with semi‐structured interviews	Thematic analysis	Saudi Arabia	Geriatric nurses' perspectives of the adoption of AI in elderly care	17 nurses	Participants acknowledged AI's potential for improving diagnostic accuracy, personalised care, continuous monitoring, and data pattern insights. However, concerns were raised regarding workflow integration, cost barriers, resistance to change, data privacy, diminishment of human elements, and the need for ethical guidelines.
Barwise et al. (2024)	A qualitative study with semi‐structured interviews	Grounded theory principles with inductive and deductive analysis methods	USA	Stakeholder perspectives of the perceived risks and benefits of using AI to identify patients with complex medical needs and language barriers	8 nurses out of a total of 48 healthcare professionals	Key perceived risks included concerns about transparency, accuracy, redundancy, privacy, perceived stigmatisation among patients, alert fatigue, and supply–demand issues. Key perceived benefits included increased awareness of in‐person interpreters, improved standard of care and prioritisation for interpreter utilisation; a streamlined process for accessing interpreters, empowered clinicians, and potential to overcome clinician bias.
Chen et al. (2021)	A qualitative empirical study with semi‐structured individual interviews and focus group	Analytic approach combining inductive and deductive coding	United Kingdom	Radiology professionals' knowledge, awareness and attitudes towards AI	6 radiographers and 12 radiologists	Radiologists had greater knowledge of the potential applications of AI than radiographers. Ongoing workforce shortages were identified as a significant issue in the adoption of AI.
Çitil and Çitil Canbay (2022)	A qualitative case study with semi‐structured interviews	Descriptive content thematic analysis	Turkey	Midwives' opinions on the future of AI and midwifery	18 midwives	Midwives remained distant and sceptical of the idea of robotic technology. Rather than seeing AI or robotic technology as part of the team, midwives envisioned that it could be integrated into midwifery care under certain conditions.
Constantin et al. (2023)	A qualitative study with semi‐structured interviews	Thematic analysis	United Kingdom	Optometrists' and ophthalmologists' attitudes to changes in the use of retinal images and preparation to use AI‐powered assistance	18 optometrists and 5 ophthalmologists	Five themes came from the analysis: Changes to professional working patterns, envisaging the image repository's impact, benefits from AI decision support, paths to improved eyecare, and education and training.
Creed et al. (2022)	A mixed‐methods study with semi‐structured focus groups, online survey and qualitative measures	Inductive coding and descriptive statistics	USA	How community mental health therapists and clinical leadership perceive AI‐ based automated evaluation and assessment as a supervision tool to provide feedback on cognitive‐behavioural therapy fidelity	30 mental health care therapists, 8 supervisors and 4 participants with other clinical leadership	Community providers perceive an AI‐based supervision platform for CBT to be acceptable, appropriate, and feasible. While perceptions of the tool were overall positive, participants raised questions and concerns that should guide future tool development and strategies for its implementation.
Dlugatch et al. (2024)	A qualitative study with semi‐structured interviews	Thematic analysis	United Kingdom	Perspectives of obstetricians and midwives regarding the ethical and trust‐related issues of incorporating AI‐driven cardiotography in the intrapartum care	5 midwives and 8 obstetricians	Four major themes emerged from interviews: the importance of accurate and efficient risk assessments, the capacity for personalisation and individualised medicine, the lack of significance regarding the type of institution that develops technology and the need for transparency in the development process.
Jairoun et al. (2024)	A qualitative study with semi‐structured interviews	Thematic analysis	United Arab Emirates	Pharmacists' perspectives on benefits and risks of using ChatGPT in pharmacy practice	35 pharmacists	Most participants felt ChatGPT could enhance compliance, use, management, safety, adherence to medication, medication counselling, minimise medication errors, and streamline medication dispensing. However, when Chat‐GPT has limited information and relies on obsolete medication databases, it risks providing inaccurate recommendations and inadequate medication details.
King et al. (2023)	A qualitative study with semi‐structured interviews	Inductive‐deductive thematic analysis	USA	Nurses' perceptions of using AI in perioperative nursing handoffs	11 nurses	Participants were receptive to AI as a potential adjunct for postoperative handoff communication.
Metsälä et al. (2020)	A qualitative study with electronic survey	Inductive thematic analysis	Finland	Radiographer and biomedical laboratory scientists' and students' perceptions of artificial intelligence in healthcare diagnostics	10 radiographers, 12 biomedical laboratory scientists, 13 radiographer students and 7 biomedical laboratory scientist students	Student and staff perceptions of the potential of AI covered topics: relief for staff workload, patient status evaluation and hastening of care, improvement of care and diagnostics and minimisation of errors with varying stress.
Ng et al. (2022)	A qualitative study with six online focus groups	Thematic analysis	Singapore	Radiographers' knowledge, perceptions, and expectations of AI	22 radiographers	Participants had limited knowledge of AI. Perceptions were mixed, recognising its benefits in increasing efficiency and improving patient care, but also aware of its limitations in accuracy and bias.
Rogan, Firth, and Bucci (2024)	A qualitative study with semi‐structured interviews	Reflexive thematic analysis	United Kingdom	Mental health professionals' views of using digital devices to passively collect data and apply machine learning in mental healthcare	5 nurses out of a total of 15 mental health professionals	Three themes were identified: positives and negatives of digital devices, passive sensing and machine learning methods in mental healthcare, barriers to use in clinical practice and facilitators to use in clinical practice.
Rony, Kayesh, et al. (2024)	A qualitative study with semi‐structured interviews	Thematic analysis	Bangladesh	Perceptions of nursing professionals regarding the role of AI in shaping the future of healthcare	23 nurses	Ten key themes emerged including: Perceptions of AI readiness; Benefits and concerns; Enhanced patient outcomes; Collaboration and workflow; Human‐tech balance: Training and skill development; Ethical and legal considerations; AI implementation barriers; Patient–nurse relationships; Future vision and adaptation.
Rony, Numan, Akter, et al. (2024)	A qualitative study with semi‐structured interviews	Thematic analysis	Bangladesh	Nurses' perspectives on privacy and ethical concerns associated with the implementation of AI in healthcare settings	20 nurses	Six themes emerged from the research findings: Ethical dimensions of AI integration; Privacy challenges in healthcare AI; Balancing innovation and ethical practice; Human touch vs. technological progress; Patient‐centred care in the AI era; and Ethical preparedness and education.
Rony, Numan, Johra, et al. (2024)	A qualitative study with semi‐structured interviews	Thematic analysis	Bangladesh	Nurse practitioners' perceptions and attitudes towards adoption of AI in health care	37 nurses	The thematic analysis revealed nine main themes including nurse practitioners' perceptions of AI implementation, attitudes towards AI adoption, patient‐centred care and AI, quality of health care delivery and AI, ethical and regulatory aspects of AI, education and training needs, collaboration and interdisciplinary relationships, obstacles in integrating AI, and AI and health care policy.
Rony, Parvin, et al. (2024)	A qualitative study with semi‐structured interviews and focus groups	Thematic analysis	Bangladesh	Healthcare workers' concerns about artificial intelligence replacing medical professionals	8 nurses and 6 pharmacists out of a total of 33 healthcare workers	Seven key themes reflecting healthcare workers' concerns, including job security and economic concerns; trust and acceptance of AI; ethical and moral dilemmas; quality of patient care; workforce role redefinition and training; patient–provider relationships; healthcare policy and regulation.
Shawli et al. (2024)	A qualitative study with semi‐structured interviews	Thematic analysis	Saudi Arabia	Physical therapy professionals' attitudes and perceptions on the use of AI in rehabilitation	15 physical therapy professionals	Participants shared views on the potential impact of AI on rehabilitation practices, such as aiding the decision‐making process, saving time and effort of both the therapist and patients. Participants have stressed potential pitfalls that still need to be considered, such as patient data privacy, potential loss of patient‐healthcare practitioner relationship, ethical concerns regarding over‐reliance on these applications and how that might hinder effective patient care.
Strauss et al. (2023)	A qualitative study with semi‐structured interviews	Inductive coding and constant comparison analysis	USA	Providers' perceptions of AI‐CDS for liver transplant listing decisions	4 advanced practice providers out of a total of 53 multidisciplinary liver transplant providers	Analysis yielded 6 themes important for the design of fair AI‐CDS for liver transplant listing decisions: transparency in the creators behind the AI‐CDS and their motivations; understanding how the AI‐CDS uses data to support recommendations; acknowledgment that AI‐CDS could mitigate emotions and biases; AI‐CDS as a member of the transplant team, not a replacement; identifying patient resource needs; and including the patient's role in the AI‐CDS.
Su et al. (2022)	An empirical interview study	Thematic analysis	USA	Healthcare providers' visions of future technologies to support their PGHD use	5 nurses out of a total of 20 healthcare professionals	The perceived challenges associated with the envisioned future, such as AI‐driven data inequity, added burden, lack of trust towards AI, and fear of being replaced by AI.
Sujan et al. (2022)	A qualitative study with semi‐structured interviews	Thematic analysis	United Kingdom	Different stakeholders' perceptions of safety and safety assurance of healthcare AI	4 nurses and 2 pharmacists out of a total of 26 stakeholders	Participant perceptions related to the potential impact of healthcare AI, requirements for human‐AI interaction, safety assurance practices and regulatory frameworks for AI and the gaps that exist, and how incidents involving AI should be managed
Turchioe et al. (2024)	A mixed‐methods study with qualitative interviews and quantitative surveys	Descriptive statistics and thematic analysis	USA	Nursing informaticists' perspectives on key challenges and opportunities for the nursing profession in the era of AI‐enriched healthcare delivery	52 nurse informatics	Four themes emerged during the qualitative analysis: current AI use among nurses; envisioning the future of nurses and AI; investing in AI implementation and preparing the nursing workforce for AI.
Wieben et al. (2024)	A qualitative study with semi‐structured interviews	Thematic analysis	USA	Nurses' perspectives on Machine Learning Clinical Decision Support (ML CDS) design, development, implementation, and adoption	17 nurses	Four major themes and 14 sub‐themes highlight nurses' perspectives on autonomy in decision‐making, the influence of prior experience in shaping their preferences for use of novel CDS tools, the need for clarity in why ML CDS is useful in improving practice/outcomes, and their desire to have nursing integrated in design and implementation of these tools.
Yoo et al. (2023)	A qualitative study with semi‐structured interviews	Content analysis	South Korea	Physicians' and nurses' needs, expectations and concerns related to medical AI in critical care settings	7 nurses and 8 physicians	Participants expressed expectations that medical AI could enhance their patients' outcomes, increase work efficiency, and reduce hospital operating costs. They also mentioned concerns regarding distortions in the workflow, deskilling, alert fatigue, and unsophisticated algorithms.
Yousif et al. (2024)	A qualitative exploratory study with semi‐structured interviews	Inductive thematic analysis	Pakistan	Healthcare professionals' perspectives on AI	6 pharmacists, 5 nurses and 14 doctors	Healthcare professionals have a positive perception regarding AI. The challenges hindering the incorporation of AI can be solved by awareness, proper training sessions, specifying extra budget for the healthcare sector and promoting research on AI.

Findings, along with illustrative quotations from the original studies, were categorised based on their similarity in meaning. Each finding was assessed for its level of credibility, and only unequivocal (U = the findings are accompanied by a contextually rooted, detailed, rich and clearly associated illustration) or credible (C = the findings are accompanied by an illustration lacking detail, richness or clear association with it) findings were included in the synthesis. Findings that did not have an illustrative quotation (Amin et al. 2025) were excluded from the meta‐aggregation and were not included in the analysis. Still, results were described narratively in the results section. The categories were named to reflect their content. These categories were then used to develop synthesised findings that reflect the core meaning of each group.

### 3.7 Synthesis

The data were pooled and synthesised using a meta‐aggregation approach. Meta‐aggregation is a method for synthesising qualitative research findings by organising them into categories and synthesising findings based on similarities in meaning. It aims to provide a detailed understanding of the phenomenon's experiences to comprehend its meanings and potentially derive practical implications (Porritt et al. 2024).

## 4 Results

### 4.1 Description of the Studies

The characteristics of the included 26 studies in the review are presented in Table 2. The original studies in the review were published between 2020 and 2025 and were conducted in Bangladesh (*n* = 4), Egypt (*n* = 1), Finland (*n* = 1), Pakistan (*n* = 1), Saudi Arabia (*n* = 2), Singapore (*n* = 1), South Korea (*n* = 1), Turkey (*n* = 1), USA (*n* = 7), the United Arab Emirates (*n* = 1) and the United Kingdom (*n* = 6).

Participants in the included studies were nurses (*n* = 14) (Amin et al. 2025; Badawy and Shaban 2025; Barwise et al. 2024; King et al. 2023; Rogan, Firth, and Bucci 2024; Rony, Kayesh, et al. 2024; Rony, Numan, Akter, et al. 2024; Rony, Numan, Johra, et al. 2024; Rony, Parvin, et al. 2024; Su et al. 2022; Sujan et al. 2022; Wieben et al. 2024; Yoo et al. 2023; Yousif et al. 2024), radiographers (*n* = 4) (Akudjedu et al. 2023; Chen et al. 2021; Metsälä et al. 2020; Ng et al. 2022), pharmacists (*n* = 4) (Jairoun et al. 2024; Rony, Parvin, et al. 2024; Sujan et al. 2022; Yousif et al. 2024), midwives (*n* = 2) (Çitil and Çitil Canbay 2022; Dlugatch et al. 2024), optometrists (*n* = 1) (Constantin et al. 2023), mental healthcare therapists (*n* = 1) (Creed et al. 2022), biomedical laboratory scientists (*n* = 1) (Metsälä et al. 2020), advanced practice providers (*n* = 1) (Strauss et al. 2023), nurse informatics (*n* = 1) (Turchioe et al. 2024) and physiotherapists (*n* = 1) (Shawli et al. 2024). Four studies included multiple healthcare professional groups (Rony, Parvin, et al. 2024; Sujan et al. 2022; Yousif et al. 2024; Metsälä et al. 2020).

### 4.2 Methodological Quality

The 26 studies were reviewed for methodological quality using JBI evaluation criteria for qualitative research (Lockwood et al. 2015). One study (Rogan, Firth, and Bucci 2024) met all 10 critical appraisal criteria, six studies (Amin et al. 2025; Badawy and Shaban 2025; Jairoun et al. 2024; Ng et al. 2022; Rony, Numan, Akter, et al. 2024; Yoo et al. 2023) met nine criteria, six studies (Akudjedu et al. 2023; Chen et al. 2021; Creed et al. 2022; Rony, Numan, Johra, et al. 2024; Rony, Parvin, et al. 2024; Sujan et al. 2022) met eight criteria, nine studies (Çitil and Çitil Canbay 2022; Constantin et al. 2023; Dlugatch et al. 2024; Turchioe et al. 2024; Rony, Kayesh, et al. 2024; Shawli et al. 2024; Strauss et al. 2023; Wieben et al. 2024; Yousif et al. 2024) met seven criteria, and four studies (Barwise et al. 2024; King et al. 2023; Metsälä et al. 2020; Su et al. 2022) met six criteria. The assessment of the methodological quality of included studies is presented in Table 3.

**TABLE 3:** Assessment of methodological quality of included studies (*n =* 26).

Study	Q1	Q2	Q3	Q4	Q5	Q6	Q7	Q8	Q9	Q10	Total
Akudjedu et al. (2023)	N	Y	Y	Y	Y	N	N	Y	Y	Y	8/10
Amin et al. (2025)	Y	Y	Y	Y	Y	Y	Y	N	Y	Y	9/10
Badawy and Shaban (2025)	Y	Y	Y	Y	Y	N	Y	Y	Y	Y	9/10
Barwise et al. (2024)	N	Y	Y	Y	Y	N	N	Y	N	Y	6/10
Chen et al. (2021)	Y	Y	Y	Y	Y	N	N	Y	Y	Y	8/10
Çitil and Çitil Canbay (2022)	N	Y	Y	Y	Y	N	N	Y	Y	Y	7/10
Constantin et al. (2023)	N	Y	Y	Y	Y	N	N	Y	Y	Y	7/10
Creed et al. (2022)	N	Y	Y	Y	Y	N	Y	Y	Y	Y	8/10
Dlugatch et al. (2024)	N	Y	Y	Y	Y	N	N	Y	Y	Y	7/10
Jairoun et al. (2024)	N	Y	Y	Y	Y	Y	Y	Y	Y	Y	9/10
King et al. (2023)	N	Y	Y	Y	Y	N	N	N	Y	Y	6/10
Metsälä et al. (2020)	N	Y	Y	Y	Y	N	N	Y	N	Y	6/10
Ng et al. (2022)	N	Y	Y	Y	Y	Y	Y	Y	Y	Y	9/10
Rogan, Firth, and Bucci (2024)	Y	Y	Y	Y	Y	Y	Y	Y	Y	Y	10/10
Rony, Kayesh, et al. (2024)	N	Y	Y	Y	Y	N	N	Y	Y	Y	7/10
Rony, Numan, Akter, et al. (2024)	Y	Y	Y	Y	Y	N	Y	Y	Y	Y	9/10
Rony, Numan, Johra, et al. (2024)	Y	Y	Y	Y	Y	N	N	Y	Y	Y	8/10
Rony, Parvin, et al. (2024)	N	Y	Y	Y	Y	N	Y	Y	Y	Y	8/10
Shawli et al. (2024)	N	Y	Y	Y	Y	N	N	Y	Y	Y	7/10
Strauss et al. (2023)	N	Y	Y	Y	Y	N	N	Y	Y	Y	7/10
Su et al. (2022)	N	Y	Y	Y	Y	N	N	Y	N	Y	6/10
Sujan et al. (2022)	N	Y	Y	Y	Y	N	Y	Y	Y	Y	8/10
Turchioe et al. (2024)	N	Y	Y	Y	Y	N	N	Y	Y	Y	7/10
Wieben et al. (2024)	N	Y	Y	Y	Y	N	Y	Y	N	Y	7/10
Yoo et al. (2023)	N	Y	Y	Y	Y	Y	Y	Y	Y	Y	9/10
Yousif et al. (2024)	N	Y	Y	Y	Y	N	N	Y	Y	Y	7/10

*Note:* JBI Critical appraisal checklist for qualitative research: Q1 = Is there congruity between the stated philosophical perspective and the research methodology? Q2 = Is there congruity between the research methodology and the research question or objectives? Q3 = Is there congruity between the research methodology and the methods used to collect data? Q4 = Is there congruity between the research methodology and the representation and analysis of data? Q5 = Is there congruity between the research methodology and the interpretation of results? Q6 = Is there a statement locating the researcher culturally or theoretically? Q7 = Is the influence of the researcher on the research, and vice‐ versa, addressed? Q8 = Are participants, and their voices, adequately represented? Q9 = Is the research ethical according to current criteria or, for recent studies, and is there evidence of ethical approval by an appropriate body? Q10 = Do the conclusions drawn in the research report flow from the analysis, or interpretation, of the data?

Abbreviations: No, N; Yes, Y.

### 4.3 Healthcare Professionals' Perceptions of AI in Healthcare

A total of 25 studies were included in the meta‐aggregation. One study (Amin et al. 2025) was excluded from meta‐aggregation because it did not include any illustrative quotations. A total of 185 findings were identified from the included studies that answered the research question. Findings were aggregated into 33 categories and then into five synthesised findings as follows: (1) Perceived benefits of AI in healthcare; (2) Perceived impact of AI on professional roles and workforce dynamics; (3) Perceived impacts of AI in communication and interaction; (4) Perceived challenges of AI related to technical, financial and systemic factors; (5) Perceived ethical, cultural and regulatory considerations regarding the use of AI. The findings, illustrations and level of credibility are presented in Table 4, and the data synthesis of the findings into categories and the synthesised findings are presented in Table 5.

**TABLE 4:** Findings, illustrations and level of credibility.

Study	Findings	Illustrations	Level of credibility^a^
Akudjedu et al. (2023)	Enhanced patient care	‘Opportunity to enhance practice, give over routine tasks to AI and for us to spend more quality time with patients in patient care and support ‐ opportunity to better serve our patients’ (Clinical practicing radiographer, p. 111)	U
Akudjedu et al. (2023)	Enhanced technology	‘It may assist in diagnosis, and I can see it being very useful for record‐keeping and being patient history’ (Clinical practicing radiographer, p. 111)	U
Akudjedu et al. (2023)	Enhanced workflow	‘It provides an opportunity for radiographers to change how they work and the services that they provide. An aim should be to improve the efficiency and accuracy of diagnosis, reducing any waits in the system and allowing prompt diagnosis so that people don't have to wait long periods of time for a diagnosis or treatment.’ (Clinical practicing radiographer, p. 110)	U
Akudjedu et al. (2023)	Professional de‐skilling	‘Dependence on the AI program all the time might lead to incompetence as they are not trying hard to learn’ (Clinical practicing radiographer, p. 112)	U
Akudjedu et al. (2023)	Uncertainty in workforce	‘Unemployment –as every organization is looking to replace the minimum qualified individuals with AI robots which can do similar work with much more efficiency’. (Clinical practicing radiographer, p. 112)	U
Badawy and Shaban (2025)	Addressing potential data breaches	‘While AI offers many benefits, we need to address the potential for data breaches and misuse of patient information’. (P11, p. 44)	U
Badawy and Shaban (2025)	Adjustments and time management	‘Integrating AI in to our workflows requires adjustments and time, which can be overwhelming with our existing workload’. (P13, p. 45)	U
Badawy and Shaban (2025)	Balancing AI use with human interaction	‘AI can take over routine tasks, freeing us up for more complex, hands‐on care’. (P2, p. 45)	C
Badawy and Shaban (2025)	Balancing technology and human care	‘We must keep the human touch in patient interactions even while we use more technology’. (P8, p. 45)	U
Badawy and Shaban (2025)	Enhanced data analysis	‘AI can provide insights that we might miss, helping us to see patterns and trends in patient data’. (P14, p. 45)	U
Badawy and Shaban (20255)	Enhanced monitoring and proactive care	‘AI can monitor patients' vital signs continuously, alerting us to any issues before they become critical’. (P4, p. 45)	U
Badawy and Shaban (2025)	Financial barriers	‘The cost of implementing AI systems is too high, especially in public healthcare settings’, (P3, p. 46)	U
Badawy and Shaban (2025)	Focus on patient care	‘AI can help reduce the administrative burden, allowing us to focus more on patient care’. (P11, p. 45)	U
Badawy and Shaban (2025)	Improved early detection	‘AI systems can analyse patient data more quickly and accurately than we can, which helps in identifying health issues before they become severe’. (P1, p. 44)	U
Badawy and Shaban (2025)	Maintaining human elements in care	‘It's important for maintaining the patient–nurse relationship, ensuring AI supports rather than replaces us’. (P10, p. 45)	C
Badawy and Shaban (2025)	Navigating AI recommendations	‘It can be difficult to navigate sometimes when AI recommendations contradict our clinical judgement’. (P9, p. 45)	U
Badawy and Shaban (2025)	Need for clear ethical standards	‘We need clear guidelines on how to ethically use AI in our practice, especially regarding patient consent and data use’, (P10, p. 46)	U
Badawy and Shaban (2025)	Regulatory and policy requirements	‘To ensure the ethical application of AI in healthcare, appropriate policies must be implemented’. (P10, p. 46)	U
Badawy and Shaban (2025)	Resistance to AI adoption	‘There is a fear that AI could eventually replace human jobs, which creates resistance among some staff’. (P17, p. 45)	U
Badawy and Shaban (2025)	Tailoring treatment plans	‘AI can help tailor treatment plans to individual patients, considering their unique health profiles’. (P6, p. 45)	U
Badawy and Shaban (2025)	Training and knowledge gaps	‘We need proper training to understand and use AI tools effectively. Without this, there's a risk of misinterpretation and errors’. (P2, p. 45)	U
Barwise et al. (2024)	Empower and support bedside team	‘I think a prompt would be helpful because sometimes, as the bedside team, I can get wrapped up in a busy morning and not always anticipate that (a patient needs an interpreter). Maybe I realise, “Oh, we're gonna have a goal of care,” but not always have the mental capacity even to put that into the forefront of my mind. That's not always my biggest priority. Sometimes there's medical things that take precedent, but maybe even having that reminder could be helpful’. (RN 05, p. 615)	U
Barwise et al. (2024)	Improved prioritisation and resource allocation for patients	‘By proactively finding these patients and providing these resources earlier on, I believe would be beneficial, not only in the care that we provide as a hospital but also the way that the patient interprets that care that they are receiving’. (RN 04, p. 615)	U
Barwise et al. (2024)	Privacy and Confidentiality	‘I just don't know exactly what is being done with that information’. (RN 09, p. 614)	U
Barwise et al. (2024)	Redundancy of AI in this context	‘I feel my assessment on whether a patient qualifies for an interpreter is more than… is better than some algorithms’ (RN 11, p. 614)	U
Chen et al. (2021)	Attitudes towards AI innovations	‘I still have my doubts about how sensitive technology can be and you really have to prove it to me before I actually accept it… You need the data to prove that there is a worthwhile system to start’. (Unit 1, Radiographer 1, p. 5).	U
Chen et al. (2021)	Knowledge of AI innovations	‘I am aware that there's quite a lot going on, because obviously I've got colleagues already involved in AI, someone like Ross (anonymised name)’ (Unit 3, Radiographer 4, p. 5).	U
Çitil and Çitil Canbay (2022)	Human competences	‘Our job is a profession that proceeds emotionally and should have humanistic values like fidelity and compassion. The use of artificial intelligence can do anything which is coded. But it does the same routines all the time. The pregnant women will not be satisfied with this as it does not give anything emotionally’. (M5, p. 1517)	U
Çitil and Çitil Canbay (2022)	Perceived deficiencies	‘Midwifery care requires an emotional bond. How machines connect with patients!’ (M4, p. 1517)	U
Çitil and Çitil Canbay (2022)	Potential advantages and conditional acceptance	‘It allows us to focus on the patient by decreasing our workload if it is used as a support in midwifery care’. (M4, p. 1516)	U
Çitil and Çitil Canbay (2022)	The future of midwifery and care	‘Artificial intelligence can functionally replace midwives, but never emotionally. The naturalness of delivery deteriorates, instrumental and caesarean rates increase’. (M5, p. 1519)	U
Çitil and Çitil Canbay (2022)	Trust issues	‘Who will individuals sue for service from a machine with unexpected damages? It is important to protect the safety of the work team and patients. Will there be a law adapted to artificial intelligence?’ (M12, p. 1518)	U
Constantin et al. (2023)	Benefits From AI Decision Support	‘One of the big things now is with ever‐increasing imaging you've got an ever‐increasing burden of reviewing…If AI allows you to do that at a more efficient pace and a more accurate pace’. (P2, p. 6)	U
Constantin et al. (2023)	Paths to Improved Eye Care	‘Often, we'll see patients … for follow‐up appointments just because we're uncertain. But if these AI technologies … even in those grey or uncertain patient situations meant we could make a better or a quicker judgement, then that would be handy’. (P18, p. 6)	U
Creed et al. (2022)	Cultural diversity	‘Yeah, and what is empathy across cultures?’ (T, p. 348)	U
Creed et al. (2022)	Privacy	‘I could see people getting access to it who are not the [client]… and to me that's a huge confidentiality risk.’ (T, p. 348)	U
Creed et al. (2022)	Rapport	‘Every relationship is different. How will it know if my client thinks I get them?’ (T, p. 348)	U
Dlugatch et al. (2024)	Accurate and efficient risk assessments	‘Like something that wasn't previously picked up on, being picked up on. Which then resulted in a healthy, a healthy delivery’. (Midwife2, p. 4).	U
Dlugatch et al. (2024)	Personalization and individualised medicine	‘So, I really strongly feel, and the research supports it, it's what I'm doing my PhD on, is continuity of midwifery care, relationship‐based care, getting to know one person or, you know, maybe two midwives. We know that it improves people's outcomes, and we don't really know why. And, like, technology can't improve that. They can't improve you feeling connected to someone. They can't improve you just noticing something slightly different about a person. And maybe acting on your instinct, which isn't quantifiable at all’. (Midwife1, p. 5)	U
Dlugatch et al. (2024)	Transparency in the development process	‘It's more around, not who, it's the what process, what ethics, what cohort, what population size, how is it conducted? [….] so, it's more around the how transferable is it? How robust is it basically? The why, it's the how did you get the results that you've got and how valid are they?’ (Midwife4, p. 7)	U
Jairoun et al. (2024)	Communication and Language Barriers	‘Insufficient awareness of cultural differences impacting communication’. (P21, p. 5)	U
Jairoun et al. (2024)	Dependency on Technology	‘Technical glitches or downtime impacting access to vital medication data’. (P29, p. 5)	U
Jairoun et al. (2024)	Efficient Medication Management	‘Providing digital tools or applications to monitor medication usage and adherence’. (P13p. 4)	U
Jairoun et al. (2024)	Enhanced Adherence to Medication Protocols	‘Providing patients with education about the potential side effects and providing strategies for managing them’. (P13, p. 4)	U
Jairoun et al. (2024)	Enhanced Medication Use	‘Offering alternatives for patients with allergies, sensitivities, or contraindications’ (P21, p. 4)	U
Jairoun et al. (2024)	Improved Medication Compliance	‘Ensuring medication instructions are adapted to accommodate patients' language proficiency or level of literacy’. (P5, p. 4)	U
Jairoun et al. (2024)	Improved Medication Counselling	‘Delivering personalised assistance to address concerns or misunderstandings related to medications’. (P14, p. 4)	U
Jairoun et al. (2024)	Improved Medication Safety	‘Keeping patients and healthcare providers informed about medication recalls or safety advisories’. (P4, p. 5)	U
Jairoun et al. (2024)	Inaccuracy in Medication Information	‘Limited information from sources resulting in inadequate medication details’. (P21, p. 5)	U
Jairoun et al. (2024)	Lack of Continuity of Care	‘Omission of important patient data during system changes or transfers’ (P25, p. 5)	U
Jairoun et al. (2024)	Lack of Personalised Care	‘Inability to recognise and respond to patients' emotional needs’. (P2, p. 5)	U
Jairoun et al. (2024)	Legal and Ethical Considerations	‘Difficulties in obtaining informed consent for utilising AI‐driven interactions’. (P34, p. 5)	U
Jairoun et al. (2024)	Minimised Medication Errors	‘Alerting users to potential interactions between prescribed medications’. (P9, p. 4)	U
Jairoun et al. (2024)	Patient Safety and Health Risks	‘Failure to identify potential drug interactions or contraindications’. (P1, p. 5)	U
Jairoun et al. (2024)	Reduced Patient‐ Healthcare Provider Interaction	‘Decline in patient satisfaction with AI‐driven interactions’. (P13, p. 5).	U
Jairoun et al. (2024)	Streamlined Medication Dispensing	‘Providing guidance on selecting and using over‐the‐counter medications appropriately’. (P33, p. 4)	U
King et al. (2023)	AI adds problems to assessment, discuss in handoff	‘If you see that these people are at a risk for an AKI or something, I would be more apt to ask about the urine output or their last [hemato]crit values. Or if it says they're at a higher risk for being admitted to the ICU, I'll be like, “Well, are they doing okay? Do they seem okay to you?” I'll be asking some questions if I see that they're at risk for these things’. (N8, Supplement 5)	C
King et al. (2023)	AI could help find relevant data	‘If your scores were all low for all your risk assessments, I probably wouldn't even try and dive deeper into that. But if they were high, somewhere you could find more information on why they were high like, “Why does this patient score so high?” either in a flowsheet … or some sort of note’. (N6, Supplement 5)	U
King et al. (2023)	AI flags easy to ignore	‘If it was a popup message, it probably wouldn't be as nice to have than if it's on the side … because sometimes stuff just pops up all the time on the computer, and … as bad as it sounds, you just exit out of it because, usually, it's something that you just haven't charted’. (N3, Supplement 5)	C
King et al. (2023)	AI identified problems/risks could lead to changes in team communication	‘I'll be getting a report from PACU, and… I'll be like, “I don't know. This patient doesn't seem like they're going to do very well on the floor. It seems they need to be monitored closely”. And sometimes you express your concerns to a doctor or a charge nurse, and they'll just be like, “Oh, well, we'll see how they do when they get here.” … maybe if they had some sort of kind of solid, concrete evidence right there in the chart … where some of these things that are a little bit harder to all put together in a firm way’ (N3, Supplement 5)	U
King et al. (2023)	AI leads to better understanding of disease severity	‘I think a red would be recurrent exacerbations, where somebody's ended up in the ER, ICU, or even just hospitalised because of a breathing issue. … or syncopal episodes that have had a fall or heart rate drop’. (N3, Supplement 5)	C
King et al. (2023)	AI problems could lead to prioritisation in monitoring and assessment	‘I would hope so. … [be]cause the patient still should be a little more closely monitored even when they're up on the floor, so I feel like it would help the floor nurse to narrow down what she should really be monitoring for’. (N7, Supplement 5)	U
King et al. (2023)	AI risks predictions could trigger specific nursing interventions	‘I would definitely say delirium would be a good one. We do see a lot of that. And we also see a lot of alcohol and drug withdrawal but was either not talked about or just unexpected. Especially for those patients, if we could monitor them with our CIWA protocol or … already have the Ativan and all the medications ordered, then we could prevent an acute event from happening or an emergency’. (N4, Supplement 5)	U
King et al. (2023)	AI triggers reassessment of important problems	‘I think it makes you more present to the physical patient as well. Looking at them and looking at all their vital signs, their trends. And I think it might just make you a little bit more mindful rather than being focused so much on charting’. (N5, Supplement 5)	U
King et al. (2023)	Easy for AI to info overload	‘Less is more. Not too much of a thing. I feel like the heavier it is, the more people are going to be like, “Oh, I don't have time for that.”’ (N4, Supplement 5)	U
Metsälä et al. (2020)	Käsitykset tekoälyn käytön mahdollisuuksista	‘Tekoälyn avulla voitaisiin tehdä tarkempia diagnooseja sekä käsitellä muutenkin helpommin suuria datavirtoja, jolloin tutkimuksen tekeminen ja terveydenhuollon innovaatiot helpottuisivat’. (Professional, p. 8)	U
Metsälä et al. (2020)	Käsitykset tekoälystä käsitteenä	‘Tekoäly toimisi henkilökunnan rinnalla ja edistäisi potilaan saamaa hoitoa’. (Professional, p. 7)	U
Metsälä et al. (2020)	Tekoälyn käytön haasteet	‘Haasteeksi voi tulla myös se, pystyykö tekoälyn analysoimaan tietoon luottaa?’ (Professional, p. 9)	U
Metsälä et al. (2020)	Tekoälyyn liittyvät eettiset näkökulmat	‘Ihmisen pitäisi kuitenkin aina arvioida potilaan tilanne kokonaisuutena ja vetää pitkälle menevät johtopäätökset ja tehdä lopullinen hoitopäätös yhteisymmärryksessä potilaan kanssa’. (Professional, p. 10)	U
Ng et al. (2022)	Knowledge of AI and its applications	‘I think AI is a big topic. It's not what we think. For example, how do you build AI involving machine learning (and) neural network? (This) requires big data’. (Rg_17, p. 557)	U
Ng et al. (2022)	Perceptions on the use of AI in radiographic practice	‘If you can identify patients that really need the scans…(we) can utilise our manpower appropriately to prioritise those examinations that require the radiographers’. (Rg_20, p. 558)	U
Ng et al. (2022)	Prospective applications and expectations of AI	‘Clinical decision support…would really help us in terms of ensuring that all the requests are appropriate. …(It) could be workflow‐based, like how we can optimise our appointments or scan protocolling’. (Rg_20, p. 559)	U
Reading Turchioe et al. (2024)	Preparing the nursing workforce for AI	‘The most successful sites that have brought AI into nursing practice have introduced it as a mentor that's intended to give you a clinical pause, but you still need to be a professional nurse. I don't know that most nurses are coming out of school right now [understand this].’ (Participant 18, p. 226)	U
Rogan, Firth, and Bucci (2024)	Abandoning Service Users and Creating Inequalities	‘Because the risk of it is that people could feel abandoned to devices, a bit like robot world’ (Participant 12, p. 6)	C
Rogan, Firth, and Bucci (2024)	Barriers for Staff	‘Erm we're short staffed, we're under pressure anyway. So, we're a bit in the negative zone at the minute, it's like well, when the hell am I going to do that? When can I do that?’ (Participant 10, p. 10)	U
Rogan, Firth, and Bucci (2024)	Facilitators for Staff	‘You could say well you know look there's evidence here that it really helps, that it's beneficial, there's been a study done and if you can provide evidence to say that it's… it has been helpful, I think erm team members would maybe be more supportive of it’. (Participant 9, p. 11–12)	U
Rogan, Firth, and Bucci (2024)	Improved Awareness and Access to Support	‘We can identify when to intervene at a much earlier stage, which has lots of positives, positives for the patient, it could keep them out of hospital, you know, it could identify a medication review much earlier or you know simply support that needs to be put in place’. (Participant 6, p. 8)	U
Rogan, Firth, and Bucci (2024)	Improved Data Collection Requiring Less Effort	‘But yeah, it would be helpful, because obviously all our clients don't tell us the truth, do they?’ (Participant 10, p. 7)	C
Rogan, Firth, and Bucci (2024)	Lack of Supportive Infrastructure	‘I'm a bit of a negative nelly and I think it's just working for the NHS it's the experience of anything you roll out, it's just one size. It's never catered to individual teams you know that we… that we work with. It's sort of just like roll it all out and make it fit’. (Participant 10, p. 10)	C
Rony, Kayesh, et al. (2024)	AI implementation barriers	‘While the potential of AI in nursing is immense, overcoming barriers like limited resources and resistance to change is essential for successful implementation. Addressing these challenges collectively is crucial for a smooth transition’. (Participant 11, p. 9)	U
Rony, Kayesh, et al. (2024)	Benefits and concerns	‘It's genuinely invigorating to consider how AI could alleviate us from mundane tasks, freeing up nurses to concentrate on intricate patient needs and fostering more profound connections with those we care for’. (Participant 14, p. 6)	U
Rony, Kayesh, et al. (2024)	Collaboration and workflow	‘Nursing is all about teamwork and collaboration. AI, when seamlessly integrated, could become a valuable partner in our efforts, streamlining tasks and enhancing coordination among healthcare professionals’. (Participant 5, p. 7)	U
Rony, Kayesh, et al. (2024)	Enhanced patient outcomes	‘I am sure that the rapid pace at which AI can analyse and interpret medical data offers the promise of quicker insights and more immediate decision‐making in patient treatment. Mmmmm …. In critical scenarios, time truly becomes a critical factor that can mean the difference between life and death’. (Participant 7, p. 7)	U
Rony, Kayesh, et al. (2024)	Ethical and legal considerations	‘As we navigate uncharted waters with AI, safeguarding patient rights and privacy remains paramount. Beyond caregiving, we must advocate for the ethical and legal implications of AI, preserving the core values of our profession’. (Participant 3, p. 8)	U
Rony, Kayesh, et al. (2024)	Future vision and adaptation	‘To be honest, nursing care's future intertwines with AI. I believe, by embracing new technologies, challenging norms, and adapting our approach, we can collectively shape a future where innovative technologies align seamlessly with compassionate patient care’. (Participant 22, p. 10)	U
Rony, Kayesh, et al. (2024)	Human‐tech balance	‘Nursing is a rich tapestry woven with empathy, compassion, and genuine human connections. While AI can certainly offer technical support, it's our unique ability to form real connections with patients that truly sets us apart’. (Participant 19, p. 7)	U
Rony, Kayesh, et al. (2024)	Patient–nurse relationships	‘A meaningful patient–nurse relationship goes beyond technology. While AI offers data‐driven insights, our capacity to deliver compassionate care remains unparalleled and central to our role’. (Participant 21, p. 9)	U
Rony, Kayesh, et al. (2024)	Perceptions of AI readiness	‘As a nurse, I see a momentous opportunity before us – to fully embrace the incorporation of artificial intelligence into nursing care. This step forward holds the promise of elevating patient outcomes and streamlining our care processes through the power of technology’. (Participant 1, p. 5)	U
Rony, Kayesh, et al. (2024)	Training and skill development	‘The integration of AI calls for a shift in our skillset. Our dedication to acquiring the necessary expertise is key to remaining at the forefront of patient care, effectively complementing our clinical acumen with AI’. (Participant 8, p. 8)	U
Rony, Numan, Akter, et al. (2024)	Algorithmic transparency and accountability	‘Nurses take care of people, and now they also deal with tricky computer stuff ‐ secrets hidden in numbers. They use transparency like a light to make sure they're doing the right thing. In the world of healthcare and computer help, nurses are like strong protectors, making sure no one's private information gets into the wrong hands’. (R10, p. 7)	U
Rony, Numan, Akter, et al. (2024)	Customization of care plans	‘Patient‐centered care in the AI era is an orchestration of personalised solutions. I believe customization is at the core of compassion, tailoring each step of the care journey to the specific needs and preferences of the individual in our care’. (R04, p. 9)	U
Rony, Numan, Akter, et al. (2024)	Ethical challenges in AI implementation	‘AI adoption in healthcare brings a wave of change, but as nurses, our compass is ethical practice. It's not just about implementing algorithms; it's about navigating the intricate terrain of patient rights, confidentiality, and trust. The ethical challenges in AI are the hurdles we must clear for a responsible future’. (R11, p. 8)	U
Rony, Numan, Akter, et al. (2024)	Ethical guidelines and frameworks	‘I believe that as nurses using AI in healthcare, it's important to follow ethical guidelines. When we use new technology, we don't forget about what's right and wrong; instead, we make our sense of what's right even stronger. Ethical rules aren't rules that limit us; they're like bright signs showing us the way to keep our journey honest, caring, and true to the principles of our job. As we move forward with new ideas and ethics, we hold the responsibility of using AI in a way that respects patient privacy and follows the values that make healthcare special’. (R19, p. 6)	U
Rony, Numan, Akter, et al. (2024)	Informed decision‐making	‘When we use AI to help take care of patients, it's not just pressing buttons; it's like creating a story with our choices. Each decision is like a part of a story, not just finishing tasks. We look at a lot of information, making sure our choices aren't quick but a well‐thought‐out plan, following what's right. Making good decisions with AI in patient care is like telling a story where the ideas come from thinking carefully, making a tale of choices that follow the rules we believe in’. (R13, p. 6)	U
Rony, Numan, Akter, et al. (2024)	Interdisciplinary collaboration in ethics	‘Imagine AI as a helper in healthcare, changing how things work. To ensure that AI can be used in the right way, everyone needs to work together. People who understand healthcare, technology, and have ethical understanding join forces. This collaboration will build a strong commitment to always prioritise fairness and goodness. In a healthcare setting with AI, success does not only mean the technology working well; it means ensuring that we always do what is right for everyone involved’. (R06, p. 10)	U
Rony, Numan, Akter, et al. (2024)	Maintaining patient‐centred communication	‘I am certain that communication serves as the bridge connecting patients to the heart of care. However, our challenge is to ensure that every interaction reflects empathy, understanding, and a genuine commitment to the patient's well‐being’. (R12, p. 10)	U
Rony, Numan, Akter, et al. (2024)	Organisational support for ethical decision‐making	‘From my sense ….ethical decision‐making is a collective responsibility. Organisational support needs to be ensured for every decision, especially in the AI‐integrated clinical setting. Simply making policies is not enough; there is a need to foster a culture of ethical mindfulness’. (R17, p. 11)	U
Rony, Numan, Akter, et al. (2024)	Patient advocacy for privacy	‘Imagine nurses as the leaders in a play when computers help in healthcare. Each part of the play is like a promise to keep private things safe for the people they help. It's a big promise to protect the special trust between the nurse and the person they are helping’. (R14, p. 7)	U
Rony, Numan, Akter, et al. (2024)	Patient empowerment through technology	‘Nursing in the age of artificial intelligence demands a nuanced approach. Patient empowerment through technology is our guidepost. We are at the intersection of innovation and ethical practice, carving a path where cutting‐edge tools amplify patient voices without compromising the sacred trust embedded in privacy’. (R07, p. 8)	U
Rony, Numan, Akter, et al. (2024)	Professional accountability and responsibility	‘Nurses are very important when it comes to using new technology like AI in healthcare. They have to be responsible and make sure they do the right things. Keeping patients' information private is crucial, and they have to think about what is right and wrong in every decision they make about using AI. It's like a big change, and nurses have to think about how it can help patients, but also be careful about any problems it might cause’. (R08, p. 7)	U
Rony, Numan, Akter, et al. (2024)	Training programmes on ethical AI use	‘In the era of advancing technology, ethical preparedness is crucial for patient care. Training programs on ethical AI use act as the compass guiding us through complexities, ensuring that every interaction with artificial intelligence reflects our commitment to moral principles in patient management’. (R15, p. 10)	U
Rony, Numan, Johra, et al. (2024)	AI and health care inequality	‘AI has the potential to revolutionise health care, but we must be vigilant. It's like a balancing act; we need to ensure AI benefits all, rather than creating a wider gap in health care access and outcomes’ (NP6, p. 10)	U
Rony, Numan, Johra, et al. (2024)	AI and patient outcomes	‘AI's influence on patient outcomes is undeniable. It allows us to practice more precision medicine, reduce complications, and enhance patient satisfaction. It's a valuable addition to our health care toolkit’ (NP8, p. 7–8)	U
Rony, Numan, Johra, et al. (2024)	AI and preventive care	‘AI has a significant role in preventive care. It analyzes patient data to identify potential risk factors, allowing us to take proactive measures and prevent health issues’ (NP6, p. 12)	U
Rony, Numan, Johra, et al. (2024)	AI as a tool for diagnosis	‘AI isn't here to replace us; it's here to support us. It complements our expertize and helps us deliver better care by improving our diagnostic abilities’ (NP23, p. 6)	U
Rony, Numan, Johra, et al. (2024)	AI in treatment decision‐making	‘AI streamlines the treatment decision process, making it more efficient. While it suggests options, the final decision always rests with us as health care providers’ (NP35, p. 6)	U
Rony, Numan, Johra, et al. (2024)	AI training challenges	‘AI training challenges include finding time and resources for learning while managing our clinical responsibilities. Institutions must provide support for practitioners to acquire the necessary skills’ (NP32, p. 14)	U
Rony, Numan, Johra, et al. (2024)	AI‐driven continuous monitoring	‘AI‐driven continuous monitoring ensures patients are under constant observation. It's a valuable safety net, alerting us to any anomalies and allowing for timely interventions’ (NP36, p. 12)	U
Rony, Numan, Johra, et al. (2024)	AI‐enhanced care coordination	‘Care coordination is smoother and more efficient with AI. It facilitates real‐time information sharing among health care providers, leading to better patient care’ (NP14, p. 12)	U
Rony, Numan, Johra, et al. (2024)	AI‐enhanced patient communication	‘In busy clinical settings, AI is a game‐changer for patient communication. It streamlines administrative tasks and helps us focus on meaningful interactions, improving the overall patient experience’ (NP6, p. 7)	U
Rony, Numan, Johra, et al. (2024)	AI's impact on workflow	‘AI has brought efficiency to our daily workflow. It takes care of routine tasks, from documentation to scheduling, freeing up our time to focus on patient care. It's like having a skilled assistant who ensures everything runs smoothly’ (NP11, p. 7)	U
Rony, Numan, Johra, et al. (2024)	AI's role in patient engagement	‘AI empowers patients by giving them easy access to information. It's a game‐changer in making them active participants in their health journey’ (NP32, p. 11)	U
Rony, Numan, Johra, et al. (2024)	Balancing technology and human interaction	‘It's a delicate balance – using AI to streamline tasks and ensure efficient care while also preserving meaningful human interactions. Patients appreciate the blend of technology and human touch’ (NP27, p. 11)	U
Rony, Numan, Johra, et al. (2024)	Communication and explanation of AI use to patients	‘Transparency is key when it comes to AI. Patients deserve to know how it's being used in their care and the benefits it brings’ (NP33, p. 11)	U
Rony, Numan, Johra, et al. (2024)	Concerns about AI's impact	‘While AI can streamline tasks, I worry about potential job displacement. It's like we're creating a future where AI takes over, and our roles diminish. We hope that AI complements our work rather than replaces it, preserving our place in patient care’ (NP19, p. 9)	U
Rony, Numan, Johra, et al. (2024)	Efficiency and timeliness	‘With AI, we're not wasting precious minutes on administrative tasks. We can dedicate more time to patients, ensuring their needs are met promptly’ (NP25, p. 12)	U
Rony, Numan, Johra, et al. (2024)	Ensuring equity in AI use	‘Equity matters in AI adoption. We must address biases to ensure fair treatment for all patients, regardless of their background’ (NP31, p. 13)	U
Rony, Numan, Johra, et al. (2024)	Enthusiasm for AI integration	‘We've come to trust AI as a dependable partner in our health care journey. At first, we were uncertain, but now we rely on AI as a knowledgeable assistant. It complements our clinical judgement, offering valuable insights and streamlining decision‐making. AI has earned its place as a reliable health care tool, aligning seamlessly with our expertize and ultimately enhancing patient care’ (NP12, p. 9)	U
Rony, Numan, Johra, et al. (2024)	Financial constraints	‘Financial constraints are a real challenge in AI adoption. The costs associated with integration can be a barrier, but we must find cost‐effective solutions’ (NP31, p. 15)	U
Rony, Numan, Johra, et al. (2024)	Government regulations and AI	‘I believe government regulations play a pivotal role in guiding the ethical use of AI in health care. They must be agile and adaptive, capable of keeping pace with AI advancements. A strong collaboration between health care professionals and policymakers is crucial to ensure that AI is harnessed for the betterment of patient care’ (NP7, p. 16)	U
Rony, Numan, Johra, et al. (2024)	Health care institutions' AI policies	‘Health care institutions require robust AI policies that set clear boundaries and ethical standards. These policies help maintain transparency and accountability, ensuring that AI benefits both patients and providers. They should evolve with technological advancements, offering guidance for ethical AI integration’ (NP3, p. 16)	U
Rony, Numan, Johra, et al. (2024)	Improved treatment plans	‘Treatment plans are more precise and patient‐ centric with AI. It helps us make decisions based on data, improving treatment outcomes’ (NP3, p. 12)	U
Rony, Numan, Johra, et al. (2024)	Informed consent	‘Informed consent in the context of AI is a multifaceted challenge. Patients should not only understand how AI contributes to their care but also be informed about the implications and potential outcomes. We need comprehensive educational materials and conversations to empower patients to make informed choices’ (NP25, p. 13)	U
Rony, Numan, Johra, et al. (2024)	Insurance and reimbursement with AI	‘Reimbursement policies should reflect the responsible integration of AI in health care. These policies must incentivise health care providers to utilise AI for quality patient care. Fair compensation for AI adoption aligns with ethical AI practices and encourages its responsible use’ (NP23, p. 16)	U
Rony, Numan, Johra, et al. (2024)	Integration of AI in educational programmes	‘Educational institutions must adapt their curricula to include AI content. AI is transforming health care, and students should graduate with AI proficiency, ready to meet evolving health care demands’ (NP18, p. 13)	U
Rony, Numan, Johra, et al. (2024)	Integration with existing systems	‘Our systems are deeply ingrained in our practice. Integrating AI needs to be smooth to ensure minimal disruptions to patient care’ (NP29, p. 15)	U
Rony, Numan, Johra, et al. (2024)	Interactions with AI specialists	‘Collaboration with AI specialists enhances our decision‐making. Their expertize complements our clinical knowledge, ensuring patients receive the best care’ (NP1, p. 15)	U
Rony, Numan, Johra, et al. (2024)	Maintaining personal connection	‘AI can support us, but it can't replace the empathy and understanding we provide. That's what truly makes patient care special’ (NP5, p. 11)	U
Rony, Numan, Johra, et al. (2024)	Nurse practitioners' input on AI implementation	‘Incorporating nurse practitioners' insights into AI implementation is a smart move. We bridge the gap between technology and patient needs, creating a more patient‐centric AI solution’ (NP15, p. 15)	U
Rony, Numan, Johra, et al. (2024)	Nurse practitioners' willingness to adopt AI	‘Nurse practitioners are ready for AI. It's like a new ally in health care. We're open to the opportunities it brings and are excited about its potential to elevate patient care’ (NP23, p. 10)	U
Rony, Numan, Johra, et al. (2024)	Privacy and data security	‘In the age of AI, data is a powerful currency, but it's also a vulnerability. We need to be vigilant in safeguarding it, implementing encryption, access controls, and audits to protect patient information. It's not just about technology; it's about ethics and trust’ (NP10, p. 13)	U
Rony, Numan, Johra, et al. (2024)	Reduction in diagnostic errors	‘AI complements our clinical judgement by minimising diagnostic errors. It's like a safety net, reducing the likelihood of missed or incorrect diagnoses’ (NP33, p. 12)	U
Rony, Numan, Johra, et al. (2024)	Regulatory compliance	‘As nurse practitioners, we're concerned about AI's impact on regulatory compliance. It's like navigating a complex maze; we need clear guidelines to ensure AI aligns with health care regulations and standards’ (NP17, p. 9)	U
Rony, Numan, Johra, et al. (2024)	Technical barriers and compatibility	‘Technical barriers can be frustrating. Ensuring compatibility between AI systems and our existing technology is crucial to minimise hiccups’ (NP19, p. 15)	U
Rony, Numan, Johra, et al. (2024)	Training on AI implementation	‘I think learning to use AI tools should be a part of our professional development. Training is vital to maximise the benefits AI can offer in our clinical work’ (NP28, p. 13)	U
Rony, Numan, Johra, et al. (2024)	Trust in AI decision support	‘AI's role in decision support is valuable, but it doesn't replace our judgement. It's like having a knowledgeable partner who provides insights, but the final decision rests with us’ (NP20, p. 8)	U
Rony, Parvin, et al. (2024)	Acceptance of AI as a Complementary Tool	‘AI can work alongside us, enhancing our capabilities and improving healthcare. We should welcome it as a valuable companion’. (R13, p. 8)	U
Rony, Parvin, et al. (2024)	Adapting to Changing Skill Requirements	‘Our skillset is expanding to include data interpretation and AI interaction. It's essential to adapt and keep our knowledge up to date for the benefit of our patients’. (R1, p. 11)	U
Rony, Parvin, et al. (2024)	Anxiety About Job Displacement	‘We've heard stories about AI systems diagnosing patients with incredible accuracy. It's great for medicine, but what about our jobs? I fear I'll become obsolete’. (FGR23, p. 7)	U
Rony, Parvin, et al. (2024)	Belief in AI's Capabilities	‘I'm amazed at what AI can do. Its diagnostic accuracy and data analysis capabilities are incredible. We should embrace it as a valuable tool’. (FGR21, p. 8)	U
Rony, Parvin, et al. (2024)	Communication Challenges with AI	‘Our challenge is ensuring that AI communicates effectively with patients, understanding their needs and providing accurate information without creating confusion or anxiety’. (R6, p. 11)	U
Rony, Parvin, et al. (2024)	Concerns About Diagnostic Accuracy	‘We've seen AI's diagnostic potential but concerns about its limitations persist. We need to ensure it doesn't lead to incorrect treatment decisions for patients’. (R6, p. 9)	U
Rony, Parvin, et al. (2024)	Distrust in AI's Decision‐Making	‘Distrust in AI's decision‐making is a real issue. We need transparency and accountability in how algorithms make clinical decisions’. (FGR32, p. 8)	U
Rony, Parvin, et al. (2024)	Ethical Decision‐Making in AI Implementation	‘I belief the ethical dimensions of AI in healthcare are intricate. We need a systematic approach to decision‐making that safeguards patients and maintains trust in our profession’. (FGR26, p. 9)	U
Rony, Parvin, et al. (2024)	Impact of AI on Patient Outcomes	‘I'm cautiously optimistic about AI's impact. It can augment our decision‐making, leading to better patient outcomes. But we need to ensure it doesn't compromise the human element of care’. (R9, p. 9)	U
Rony, Parvin, et al. (2024)	Legal Implications of AI in Healthcare	‘As healthcare professionals, we can't ignore the legal aspects of AI. It's essential to understand the implications and advocate for policies that protect both patients and practitioners’. (FGR15, p. 11)	U
Rony, Parvin, et al. (2024)	Moral Concerns About Replacing Medical Professionals	‘Moral concerns weigh heavily on me. The idea of AI taking over patient care raises questions about the human touch, empathy, and the doctor–patient relationship’. (FGR32, p. 8)	U
Rony, Parvin, et al. (2024)	Preparing the Workforce for AI Changes	‘We must prepare the workforce by encouraging a proactive approach to AI. We can't resist change; we need to embrace it and make it work for us and our patients’. (R2, p. 10)	U
Rony, Parvin, et al. (2024)	Redefining Healthcare Roles in the AI Era	‘We're at a crossroads in healthcare. AI is reshaping our roles. We're no longer just caregivers; we're becoming data interpreters and technology experts, which is a big shift’. (R13, p. 10)	U
Rony, Parvin, et al. (2024)	Regulatory Frameworks for AI in Healthcare	‘Establishing strong regulatory frameworks for AI in healthcare is vital. We need clear guidelines to ensure patient safety and quality standards in an AI‐driven healthcare landscape’. (R2, p. 11)	U
Rony, Parvin, et al. (2024)	Strategies to Enhance Patient Care with AI	‘In our hospital, the strategy is to embrace AI for tasks that can be automated, so we can spend more quality time with patients, focusing on their unique needs and concerns’. (FGR24, p. 9)	U
Rony, Parvin, et al. (2024)	Technological Unemployment	‘The fear of technological unemployment is widespread in our profession. It's time to have a comprehensive plan for the future’. (FGR28, p. 7)	U
Rony, Parvin, et al. (2024)	Training Needs for AI Integration	‘Training is essential to harness the power of AI. We must equip healthcare professionals with the knowledge and skills required to use these tools effectively’. (FGR25, p. 10)	U
Shawli et al. (2024)	AI and the fast‐approaching future	‘By the time future generations are coming into practice, AI might be in full form, so we have to make them aware of the latest developments’ (Participant 4, p. 5)	U
Shawli et al. (2024)	AI as a decision maker	‘In case of prescription and diagnosis and setting goals and everything artificial intelligence will explore better than us [Therapists]’ (Participant 10, p. 4)	U
Shawli et al. (2024)	Ethical concerns and potential pitfalls	‘We cannot completely depend on that [AI applications] because some errors can happen, so there needs to be human interventions, the machine is a machine always’ (Participant 4, p. 5)	U
Shawli et al. (2024)	Factors influencing implementation	‘[we need to anticipate] load on the software, shutdowns, technical problems’ (Participant 6, p. 5).	U
Shawli et al. (2024)	Maintaining the therapist‐to‐patient interaction is vital	‘We cannot leave the patient [alone] to deal with this device, it's a device even if it's intelligent, we [as clinicians] need to be in contact with the patient’ (Participant 3, p. 5)	U
Shawli et al. (2024)	Overall understanding of AI applications	‘Artificial intelligence is just like the human mind so that it can learn, understand and give like a decision so that when we add it to a device it becomes smarter’ (Participant 9, p. 3)	U
Shawli et al. (2024)	Potential applications and benefits of introducing AI in rehabilitation	‘[it can be used to] distribute caseloads based on patient [complaints] who will actually need the priority to be seen … say for example within 24–48 h in a post operation? so you need to filter through them, I think artificial intelligence will be really good at detecting these patterns and finding the proper slot in time to be seen’ (Participant 8, p. 4)	U
Shawli et al. (2024)	Rehabilitation professionals are not easily replaced: Impact on workforce	‘We [as physical therapists], our hands are the skills, you cannot say that this is going to take the place of the physical therapist’ (Participant 7, p. 4)	U
Strauss et al. (2023)	Acknowledgment that AI‐CDS could mitigate emotions and biases	‘We're human, so I think that we have emotion, and we sometimes can allow emotions to play into our clinical decision making, and that can be a good thing, or it can be a bad thing. And sometimes I think taking that emotion or that personal experience, out of it, would occasionally potentially be a good thing…maybe it'll be better to allow a machine to make clinical decisions on patients because it takes the emotion out of it…a tool wouldn't care if you were rich or if you are poor, and a tool wouldn't care if you were black or if you were white.’ (#0208 APP, p. 6)	U
Strauss et al. (2023)	Understanding how the AI‐CDS uses data to support recommendations	‘A tool by definition meets that standard [fairness] right…if gender or race or class or any of those things don't have anything to do with what the tool is asking for, then I think that the tool by nature is not discriminatory’. (#0208 APP, p. 6)	U
Su et al. (2022)	Envisioned Challenges of PGHD‐based AI Systems	‘If you do depend on technology to tell you what to do, then you're going to lose the clinical instinct. If AI malfunctions, it can be very dangerous. There's going to be a whole generation of physicians who are like, “The AI is not working, so I'm not sure how to treat these patients.”’ (P16, p. 15)	U
Su et al. (2022)	Envisioned Future: Clinical Use of PGHD Support by AI	‘The system can set up green, yellow, and red zones. I think the system can flag a patient who has a clear level of worsening based on the data they tracked and show that this patient is in the red zone’ (P5, p. 11).	C
Su et al. (2022)	The Synergy Between Human and AI	‘I think that a good AI system should always involve us, providers, to remind patients about something. I was surprised that even my provider would do that for me. She would mail me letters just to remind me that I need to go for the follow‐up for this or that, and you know, that's always nicer than getting an automated reminder’ (P17, p. 18).	U
Sujan et al. (2022)	Advantages, disadvantages and impact on patient experience	‘With regards to things automatically going up and down, sometimes it isn't just numbers. It's what you're looking at. Does that make sense? It's not just about numbers. It's what you're looking at […] Sometimes looking at a patient you just know what they need. You don't really need anything else to tell you. You can look at a patient and you can think, oh, God, this is not going to be a good day. This is going to end badly. You know just by looking at somebody, which obviously can't be quantified’. (Staff‐01, p. 4)	C
Sujan et al. (2022)	Incident investigation	‘Who's going to be accountable for that then because would it… if your patient dies and you end up in Coroners because the noradrenaline wasn't being administered and they had no blood pressure and then they arrest. Is it good enough to say well the pump didn't tell me? It's my pin number, it's still my patient. It's accountability.’ (Staff‐04, p. 8)	U
Turchioe et al. (2024)	Current AI use among nurses	‘We created a device that [detects nurses] and we pair the signals with beacons worn on the name tag. When you fuse them using a process called edge AI, our AI detects a person moving out of bed. Also, for pressure injury prevention, the device knows if the person hasn't turned in over two hours’. (Participant 3, p. 225)	U
Turchioe et al. (2024)	Envisioning the future of nurses and AI	‘The average age of the nurses is 52. How do we improve that pipeline when we don't have people to actually do it? Could AI be able to automate processes that are currently manual to offload some of the processes that people are performing?’ (Participant 16, p. 225–226)	U
Turchioe et al. (2024)	Investing in AI implementation	‘New nurses, if they're trained and they grow up with these AI interfaces…aren't going to be the impediment. I think money and resources for successful implementation are going to be the impediments’. (Participant 17, p. 226)	U
Wieben et al. (2024)	Decision support: from seamless integration to fatiguing vexation	‘I always look at labs and vital signs, but sometimes I don't put them altogether […] I might miss something and that [CDS] kind of always makes me take a second look, like what's going on with my patient?’ (P08, p. 87)	U
Wieben et al. (2024)	Evidence of novel insights	‘Is this really moving towards something that's going to improve our ability to care for our patients?’ (P08, p. 88)	U
Wieben et al. (2024)	More than a score, guide nurses to action	‘Okay, cool. And now what?’ […] if you're not going to give me information as far as ways to help me guide my practice to mitigate that? (P12, p. 87)	U
Wieben et al. (2024)	Nurse decision‐making autonomy: Locus of control in flux	‘You can score them as a high risk and still decide […] So, I feel like that still gives us some autonomy in our decision‐making, you know, like whether we feel they actually are a high fall risk or not’. (P08, p. 87)	U
Wieben et al. (2024)	Nurses aspire to dynamic engagement with ML CDS	‘Because it can be frustrating being told things that we already know or being told things that are that patient's normal. So if they had a button that we could click and be like, this is the normal, something like that, so that it—the program could learn that patient's uniqueness’ (P09, p. 87)	U
Wieben et al. (2024)	Patient benefit realisation	‘Make it more patient‐centered to our patients. […] there're so many different types of people out here’. (P10, p. 87)	U
Wieben et al. (2024)	Technology's influence on nurse ‘brainwork’	‘It's easy to skip the like critical thinking portion of it a little bit […] because it's almost automatic’. (P01, p. 86)	U
Wieben et al. (2024)	Trust in the development team: Nurses and ‘others’	‘I would just tend to feel better when nurses are involved; I tend to be more curious about it because I really trust other nurses, and not that I don't trust other people, because we need a whole team to create these tools, but I would like to know that it was a whole team and that my discipline was really included and helped shape it. That would make me feel more confident in the tool’. (P08, p. 87)	U
Wieben et al. (2024)	What ‘Machine Learning’ means to nurses and nursing practice	‘if I just had that general knowledge of what it was encompassing, I would understand its usefulness a little bit better’ (P04, p. 89)	U
Yoo et al. (2023)	Expectations of AI Algorithms	‘The occurrence of delirium among patients will decrease. Patients will experience less pain, and, consequently, hospital stays and unplanned intubation will also decrease. Since additional drugs are administered if delirium occurs, I think it will also prevent administering unnecessary drugs’. (ICU charge nurse, p. 67)	U
Yoo et al. (2023)	Expected Responses in the Presence of a Decision that Conflicts with Medical AI	‘AI is not 100% accurate, but it has a larger volume of data than I do, doesn't it? My clinical judgement is based on my 5‐year clinical experience, so if an algorithm suggests an option conflicting with my judgement, I will seek the opinions of other medical staff members and decide to prevent complications as entirely as possible’. (ER nurse, p. 70)	U
Yoo et al. (2023)	Strategies for Successful Clinical Implementation of Medical AI	‘I think the use of AI would be terminated if there was no difference in outcomes when AI is used versus before, or if the outcomes were worse. It seems that AI implementation should start with where there will be no harm and be gradually increased if it is reasonably effective’. (ER staff nurse, p. 71)	U
Yousif et al. (2024)	Challenges in incorporation of AI with proposed solutions	‘Yes, it is possible. Machines have already replaced humans in technology. Machines have replaced humans in the industry. It is possible that in the future, it may happen in our healthcare profession as well’. (Pharmacist 06, p. 6)	U
Yousif et al. (2024)	Cognizance of AI	‘I think artificial intelligence is an ability that humans develop in computers. I don't know much about it, but I recently watched a video on YouTube’. (Nurse‐02, p. 3).	U
Yousif et al. (2024)	Merits and demerits of AI	‘It's useful; tasks that once took four hours can now be done in an hour, 30 min, or even less’. (Pharmacist‐02, p. 5)	U

^a^ Level of credibility: C, credible; NS, not supported; U, unequivocal.

**TABLE 5:** Data synthesis of findings into categories and synthesised findings.

Findings (*n =* 185)	Categories (*n =* 33)	Synthesised findings (*n =* 5)
Enhanced workflow Collaboration and workflow AI's impact on workflow Merits and demerits of AI AI‐enhanced care coordination Perceptions on the use of AI in radiographic practice Envisioning the future of nurses and AI	Improvement in workflow	**Perceived benefits of AI in healthcare** *Healthcare professionals perceived various ways in which AI could benefit their work and improve the quality of patient care*
Enhanced patient care Focus on patient care Strategies to Enhance Patient Care with AI Impact of AI on Patient Outcomes Balancing AI use with human interaction Potential advantages and conditional acceptance Benefits and concerns Efficiency and timeliness AI‐enhanced patient communication	2 Increased time for patient care	
Decision support: from seamless integration to fatiguing vexation Nurse decision‐making autonomy: Locus of control in flux Benefits From AI Decision Support AI as a decision maker Prospective applications and expectations of AI AI in treatment decision‐making Enhanced patient outcomes	3 Decision support in patient care	
Improved treatment plans Customization of care plans Tailoring treatment plans	4 Customised patient journey	
Enhanced Adherence to Medication Protocols Improved Medication Compliance Minimised Medication Errors Improved Medication Counselling Streamlined Medication Dispensing Enhanced Medication Use Efficient Medication Management Improved Medication Safety Expectations of AI Algorithms	5 Improvement in pharmacological treatment	
Enhanced data analysis AI could help find relevant data Improved Data Collection Requiring Less Effort Envisioned Future: Clinical Use of PGHD Support by AI	6 Advanced use of data	
Improved Awareness and Access to Support AI and preventive care Improved early detection Paths to Improved Eye Care Accurate and efficient risk assessments AI risks predictions could trigger specific nursing interventions	7 Early identification and prevention	
Reduction in diagnostic errors Enhanced technology Perceptions of the potential uses of artificial intelligence AI as a tool for diagnosis AI and patient outcomes	8 Enhancing diagnostic accuracy	
Improved prioritisation and resource allocation for patients AI triggers reassessment of important problems AI identified problems/risks could lead to changes in team communication AI adds problems to assessment, discuss in handoff AI leads to better understanding of disease severity Potential applications and benefits of introducing AI in rehabilitation Empower and support bedside team	9 Recognising the need of care

	Patient empowerment through technology AI's role in patient engagement	10 Patient empowerment
	Enhanced monitoring and proactive care AI problems could lead to prioritisation in monitoring and assessment AI‐driven continuous monitoring	11 Enhanced clinical monitoring
Uncertainty in workforce Resistance to AI adoption The future of midwifery and care Concerns about AI's impact Technological Unemployment Anxiety About Job Displacement Rehabilitation professionals are not easily replaced: Impact on workforce Challenges in incorporation of AI with proposed solutions	12 Changes to workforce	**Perceived impact of AI on professional roles and workforce dynamics** *Healthcare professionals perceived AI as a transformative force that changes clinical roles and work dynamics. The extent of knowledge and acceptance of AI influences the readiness to adopt AI in healthcare*
Professional de‐skilling Envisioned Challenges of PGHD‐based AI Systems Technology's influence on nurse ‘brainwork’	13 Decrease in professional competence	
Barriers for Staff AI training challenges Adjustments and time management	14 Time constraints	
Training on AI implementation Training Needs for AI Integration Training and knowledge gaps Training and skill development Integration of AI in educational programmes Adapting to Changing Skill Requirements Training programmes on ethical AI use More than a score, guide nurses to action	15 Need for training	
Preparing the nursing workforce for AI Preparing the Workforce for AI Changes AI implementation barriers Future vision and adaptation Redefining Healthcare Roles in the AI Era AI and the fast‐approaching future	16 Preparing for the change in work	
Knowledge of AI innovations Knowledge of AI and its applications Current AI use among nurses Overall understanding of AI applications What ‘Machine Learning’ means to nurses and nursing practice Cognizance of AI Perceptions of artificial intelligence as a concept	17 Knowledge of AI	
Acceptance of AI as a Complementary Tool Enthusiasm for AI integration Nurse practitioners' willingness to adopt AI Belief in AI's Capabilities Perceptions of AI readiness	18 Acceptance of AI

	Distrust in AI's Decision‐Making Trust issues Trust in AI decision support Challenges in using artificial intelligence Redundancy of AI in this context Privacy and Confidentiality Privacy	19 Lack of trust
	Facilitators for Staff Evidence of novel insights Transparency in the development process Attitudes towards AI innovations	20 Need for evidence
Balancing technology and human interaction Balancing technology and human care Maintaining human elements in care Human competences Human‐tech balance Maintaining personal connection Patient–nurse relationships Perceived deficiencies Maintaining patient‐centred communication Moral Concerns About Replacing Medical Professionals The Synergy Between Human and AI Advantages, disadvantages and impact on patient experience Personalization and individualised medicine Lack of Personalised Care Acknowledgment that AI‐CDS could mitigate emotions and biases	21 Interaction between patients and healthcare professionals	**Perceived impacts of AI in communication and interaction** *Healthcare professionals perceived the importance of maintaining human connection in technological landscape of care*
Communication and Language Barriers Communication Challenges with AI Rapport	22 Communication challenges	
Interactions with AI specialists Nurse practitioners' input on AI implementation Interdisciplinary collaboration in ethics Trust in the development team: Nurses and ‘others’	23 Multiprofessional collaboration	
Reduced Patient‐ Healthcare Provider Interaction Abandoning Service Users and Creating Inequalities Maintaining the therapist‐to‐patient interaction is vital Patient benefit realisation	24 Decrease in patient satisfaction	
Technical barriers and compatibility Ethical concerns and potential pitfalls Factors influencing implementation Dependency on Technology Lack of Continuity of Care	25 Technical problems	**Perceived challenges of AI related to technical, financial and systemic factors** *Healthcare professionals identified various challenges associated with AI implementation, including technical limitations, financial constraints and systemic barriers*
Nurses aspire to dynamic engagement with ML CDS Easy for AI to info overload AI flags easy to ignore	26 Information overload	
Inaccuracy in Medication Information Concerns About Diagnostic Accuracy Patient Safety and Health Risks Addressing potential data breaches	27 Lack of accurate data	
Integration with existing systems Lack of Supportive Infrastructure Strategies for Successful Clinical Implementation of Medical AI	28 Integrating AI into existing systems	
Financial constraints Financial barriers Investing in AI implementation	29 Financial challenges	
Need for clear ethical standards Ethical and legal considerations Legal and Ethical Considerations Ethical guidelines and frameworks Ethical challenges in AI implementation Ethical considerations related to artificial intelligence Informed consent	30 Ethical challenges	**Perceived ethical, cultural and regulatory considerations regarding the use of AI** *Healthcare professionals expressed concerns about the ethical use of AI, highlighting issues such as data privacy, cultural sensitivity and the need for clear regulatory frameworks to ensure responsible integration into healthcare*
Regulatory Frameworks for AI in Healthcare Government regulations and AI Regulatory compliance Regulatory and policy requirements Organisational support for ethical decision‐making Health care institutions' AI policies Legal Implications of AI in Healthcare Ethical Decision‐Making in AI Implementation Insurance and reimbursement with AI	31 Need for regulation and organisational support	
Algorithmic transparency and accountability Informed decision‐making Patient advocacy for privacy Professional accountability and responsibility Communication and explanation of AI use to patients Incident investigation Expected Responses in the Presence of a Decision that Conflicts with Medical AI Navigating AI recommendations Privacy and data security	32 Healthcare professionals' ethical responsibility	
Cultural diversity AI and health care inequality Ensuring equity in AI use	33 Cultural dilemmas	

#### 4.3.1 Synthesised Finding: Perceived Benefits of AI in Healthcare

The first synthesised finding included 11 categories, which reflected 62 findings. The first category, *Improvement in workflow*, was supported by seven findings. Healthcare professionals perceived that AI could handle routine tasks (Rony, Numan, Johra, et al. 2024), utilise manpower appropriately (Ng et al. 2022) and automate processes (Turchioe et al. 2024). One pharmacist noted that tasks that once took 4 h can now be completed in an hour or even less with the assistance of AI (Yousif et al. 2024). The second category, *Increased time for patient care*, was supported by nine findings. With the help of AI, healthcare professionals could spend more time with patients (Akudjedu et al. 2023), have more time on hands‐on patient care (Badawy & Shaban, 2025), and concentrate on fulfilling patients' needs (Rony, Kayesh, et al. 2024). The third category, *Decision support in patient care*, was supported by seven findings. Healthcare professionals perceived that AI would facilitate data exploration for decision‐making more effectively than professionals (Shawli et al. 2024) and more quickly in emergencies (Rony, Kayesh, et al. 2024; Constantin et al. 2023). In one study, a nurse noted that clinical decision support would still leave some autonomy in decision‐making (Wieben et al. 2024).

The fourth category, *Customised patient journey*, was supported by three findings. AI can help tailor treatment plans to individual patients, making them more precise and considering specific needs (Badawy & Shaban, 2025; Rony, Numan, Akter, et al. 2024; Rony, Numan, Johra, et al. 2024). The fifth category, *Improvement in pharmacological treatment*, was supported by nine findings. AI‐enhanced tools could help offer alternative medications in case of contraindications, monitor medication usage and alert users for potential interactions between medications (Jairoun et al. 2024). The sixth category, *Advanced use of data*, was supported by four findings. AI can help healthcare professionals identify patterns and trends in patient data (Badawy & Shaban, 2025). In one study, a nurse suggested that AI could flag a patient who shows a clear level of worsening based on the data (Su et al. 2022).

The seventh category, *Early identification and prevention*, was supported by six findings. Healthcare professionals perceived that AI could help to detect the need for intervention earlier (Rogan, Firth, and Bucci 2024), identify potential risk factors to help prevent health issues (Rony, Numan, Johra, et al. 2024), and help to pick up things that were not usually picked up on (Dlugatch et al. 2024). The eighth category, *Enhancing diagnostic accuracy*, was supported by five findings. AI was seen as a safety net that can reduce diagnostic errors (Rony, Numan, Johra, et al. 2024) and facilitate more accurate diagnoses (Metsälä et al. 2020). The ninth category, *Recognising the need for care*, was supported by seven findings. Healthcare professionals perceived that AI could proactively identify patients who need support at an earlier stage (Barwise et al. 2024), activate reassessment of important issues (King et al. 2023) and prioritise patient flow (Shawli et al. 2024).

The tenth category, *Patient empowerment*, was supported by two findings. Healthcare professionals highlighted that AI could enhance patient empowerment by increasing access to information and making them active participants in their care (Rony, Numan, Akter, et al. 2024; Rony, Numan, Johra, et al. 2024). The eleventh category, *Enhanced clinical monitoring*, was supported by three findings. Healthcare professionals perceived that AI could continuously monitor vital signs and alert before issues become critical (Badawy & Shaban, 2025; Rony, Numan, Johra, et al. 2024), as well as prioritise which aspects should be monitored (King et al. 2023).

#### 4.3.2 Synthesised Finding: Perceived Impact of AI on Professional Roles and Workforce Dynamics

The second synthesised finding included nine categories, which reflected 51 findings. The twelfth category, *Changes to workforce*, was supported by eight findings. Healthcare professionals were worried that AI could lead them to unemployment (Akudjedu et al. 2023; Badawy & Shaban, 2025; Rony, Numan, Johra, et al. 2024; Rony, Parvin, et al. 2024; Yousif et al. 2024), but some professionals believed that AI could not completely replace their expertise (Çitil and Çitil Canbay 2022; Shawli et al. 2024). The thirteenth category, *Decrease in professional competence*, was supported by three findings. Healthcare professionals have expressed concerns that dependence on AI could lead to incompetence (Akudjedu et al. 2023) and a decline in critical thinking (Wieben et al. 2024). In one study, one nurse expressed concern that future generations of physicians might become too dependent on AI, stating that some may struggle to treat patients without it (Su et al. 2022).

The fourteenth category, *Time constraints*, was supported by three findings. Healthcare professionals expressed a lack of time to learn to use AI due to their existing workload and clinical responsibilities (Rogan, Firth, and Bucci 2024; Badawy & Shaban, 2025; Rony, Numan, Johra, et al. 2024). The fifteenth category, *Need for training*, was supported by eight findings. Healthcare professionals have described the need for proper training to effectively utilise AI tools (Rony, Parvin, et al. 2024; Badawy & Shaban, 2025; Rony, Kayesh, et al. 2024). They also mentioned that training should be integrated into education so that students will know how to use it by the time they graduate (Rony, Numan, Johra, et al. 2024). The sixteenth category, *Preparing for the change in work*, was supported by six findings. Healthcare professionals perceived that preparing the workforce for AI involves adapting approaches (Rony, Kayesh, et al. 2024) and understanding the changes that AI brings to the profession (Rony, Parvin, et al. 2024). Additionally, it was noted that future generations should be informed about the latest AI developments (Shawli et al. 2024).

The seventeenth category, *Knowledge of AI*, was supported by seven findings. The overall knowledge of AI among healthcare professionals varied. While some were aware that specific AI tools were already used in healthcare (Chen et al. 2021; Turchioe et al. 2024), others expressed a desire to learn more (Wieben et al. 2024). AI was generally perceived as a significant and timely topic (Ng et al. 2022). The eighteenth category, *Acceptance of AI*, was supported by five findings. Healthcare professionals expressed that AI should be embraced as both a valuable companion and a powerful tool in healthcare (Rony, Parvin, et al. 2024). AI was perceived as bringing a momentous opportunity for professionals (Rony, Kayesh, et al. 2024).

The nineteenth category, *Lack of trust*, was supported by seven findings. Healthcare professionals raised concerns about who would be held responsible if AI causes unexpected damages (Çitil and Çitil Canbay 2022). They need transparency and accountability in how AI makes clinical decisions (Rony, Parvin, et al. 2024) and where the information comes from (Barwise et al. 2024). The twentieth category, *Need for evidence*, was supported by four findings. Healthcare professionals have expressed the need for evidence demonstrating the usefulness of AI (Rogan, Firth, and Bucci 2024; Wieben et al. 2024; Chen et al. 2021), as well as transparency regarding its development (Dlugatch et al. 2024).

#### 4.3.3 Synthesised Finding: Perceived Impacts of AI in Communication and Interaction

The third synthesised finding included four categories, which reflected 26 findings. The twenty‐first category, *Interaction between patients and healthcare professionals*, was supported by 12 findings. Healthcare professionals expressed that while AI can offer technical support in healthcare, it cannot replace the emotional connection between professionals and patients (Çitil and Çitil Canbay 2022; Rony, Kayesh, et al. 2024; Rony, Numan, Johra, et al. 2024; Jairoun et al. 2024). However, one healthcare professional has noted that removing emotion from care could have benefits, potentially making care more equal (Strauss et al. 2023). A delicate balance is needed between technology and human touch (Rony, Numan, Johra, et al. 2024; Badawy & Shaban, 2025; Sujan et al. 2022). Healthcare professionals have emphasised the importance of maintaining patient relationships as AI becomes increasingly integrated into healthcare practice (Badawy & Shaban, 2025; Rony, Numan, Akter, et al. 2024; Rony, Parvin, et al. 2024).

The twenty‐second category, *Communication challenges*, was supported by three findings. Healthcare professionals have expressed concerns about communication between AI systems and patients. They questioned AI's capability to understand the meaning of speech (Creed et al. 2022) and to provide accurate information with it without confusion (Rony, Parvin, et al. 2024). The twenty‐third category, *Multiprofessional collaboration*, was supported by four findings. Healthcare professionals emphasised the importance of collaboration with AI specialists to complement clinical knowledge to ensure that patients receive the best possible care (Rony, Numan, Johra, et al. 2024; Rony, Numan, Akter, et al. 2024). In one study, a nurse mentioned that they would feel more confident using the AI tool if they knew that nurses had been involved in its development (Wieben et al. 2024). The twenty‐fourth category, *Decrease in patient satisfaction*, was supported by four findings. Healthcare professionals perceived that the use of AI could decrease patient satisfaction (Jairoun et al. 2024). They expressed concerns that leaving patients alone with machines could lead to problems (Rogan, Firth, and Bucci 2024; Shawli et al. 2024).

#### 4.3.4 Synthesised Finding: Perceived Challenges of AI Related to Technical, Financial and Systemic Factors

The fourth synthesised finding included five categories, which reflected 18 findings. The twenty‐fifth category, *Technical problems*, was supported by five findings. Healthcare professionals expressed frustration at technical barriers (Rony, Numan, Johra, et al. 2024; Jairoun et al. 2024), fear of errors and shutdowns (Shawli et al. 2024), and losing important data during system changes (Jairoun et al. 2024). The twenty‐sixth category, *Information overload*, was supported by three findings. Healthcare professionals highlighted that an excessive amount of AI‐generated information can be easily overlooked or dismissed (Wieben et al. 2024; King et al. 2023) as it may be perceived as overwhelming or irrelevant. A more concise and targeted delivery of information was preferred (King et al. 2023).

The twenty‐seventh category, *Lack of accurate data*, was supported by four findings. It was noted that the limited information available to AI from its sources could lead to inadequate mediation details (Jairoun et al. 2024) or treatment decisions (Rony, Parvin, et al. 2024). Furthermore, concerns were raised about the potential for data breaches (Badawy & Shaban,^2025^ ). The twenty‐eighth category, *Integrating AI into existing systems*, was supported by three findings. Healthcare professionals hoped for smooth AI integration into existing systems to ensure minimal disruptions to patient care (Rony, Numan, Johra, et al. 2024). Some healthcare professionals perceived the integration as challenging, expressing concerns that a non‐tailored approach does not reflect the unique characteristics of individual healthcare teams (Rogan, Firth, and Bucci 2024). The twenty‐ninth category, *Financial challenges*, was supported by three findings. Healthcare professionals expressed that the cost of implementing AI in healthcare is too high, which is a significant barrier to AI adoption in healthcare (Badawy & Shaban, 2025; Rony, Kayesh, et al. 2024; Rony, Numan, Akter, et al. 2024; Rony, Numan, Johra, et al. 2024; Rony, Parvin, et al. 2024; Turchioe et al. 2024).

#### 4.3.5 Synthesised Finding: Perceived Ethical, Cultural and Regulatory Considerations Regarding the Use of AI

The fifth synthesised finding included four categories, which reflected 28 findings. The thirtieth category, *Ethical challenges*, was supported by seven findings. Healthcare professionals perceived the need for clear ethical guidelines for using AI (Badawy & Shaban, 2025). One ethical dilemma identified was the difficulty of obtaining informed consent for the use of AI‐driven tools (Jairoun et al. 2024; Rony, Numan, Johra, et al. 2024). From an ethical perspective, it was also emphasised that healthcare professionals should evaluate the patient's condition holistically and make final care decisions in agreement with the patient when using AI (Metsälä et al. 2020). The thirty‐first category, *Need for regulation and organisational support*, was supported by nine findings. Healthcare professionals have expressed concerns about the impact of AI on regulatory compliance (Rony, Numan, Johra, et al. 2024), emphasising the need for appropriate policies (Badawy & Shaban, 2025) and regulatory frameworks to guide its use in healthcare (Rony, Parvin, et al. 2024). Professionals underlined that successful AI adoption requires coordinated support at organisational (Rony, Numan, Akter, et al. 2024), governmental and institutional levels (Rony, Numan, Johra, et al. 2024).

The thirty‐second category, *Healthcare professionals' ethical responsibility*, was supported by nine findings. Healthcare professionals expressed a sense of responsibility to protect patient privacy (Rony, Numan, Akter, et al. 2024; Rony, Numan, Johra, et al. 2024) and to maintain transparency with patients regarding the use of AI (Rony, Numan, Johra, et al. 2024). If an AI system were to suggest an option that conflicted with their clinical judgement, they emphasised the importance of seeking opinions from colleagues to avoid potential complications (Yoo et al. 2023; Badawy & Shaban, 2025). Furthermore, it was acknowledged that even when errors occur with AI, professionals would ultimately remain accountable (Sujan et al. 2022). The thirty‐third category, *Cultural dilemmas*, was supported by three findings. Healthcare professionals expressed concerns about whether AI can acknowledge cultural differences (Creed et al. 2022). It was also mentioned that healthcare professionals must ensure AI benefits everyone rather than creating wider disparities in healthcare access and outcomes (Rony, Numan, Johra, et al. 2024).

#### 4.3.6 Findings Excluded From Meta‐Aggregation

Amin et al. (2025) investigated nurses' attitudes towards AI and its adoption in nursing. According to the qualitative data, obstacles to AI adoption included a lack of knowledge and technological challenges, such as the unavailability of a stable network. In the context of readiness for AI, the results indicated that the need for training was a pivotal factor. Some nurses completely refused AI due to the fear of losing the human touch in care. The results also showed that while some nurses acknowledged AI's potential to improve efficiency, others cited outdated knowledge as a barrier, highlighting the need for continuous education and support. Additionally, nurses expressed varying expectations regarding changes in nursing roles with the use of AI (Amin et al. 2025).

## 5 Discussion

This systematic review of qualitative studies aimed to identify the experiences and perceptions of healthcare professionals on AI in healthcare. Five synthesised findings from 25 studies were identified through meta‐aggregation, and the findings from one study were described narratively. The review highlighted that while healthcare professionals acknowledge the benefits of AI in healthcare, they also raise concerns about its impact on professional roles and workforce dynamics, the importance of preserving human connection in care, challenges related to AI implementation and ethical considerations surrounding its use.

The first synthesised finding revealed the perceived positive ways in which AI could support healthcare professionals in their roles. Key benefits included enhanced workflow efficiency and the ability to allocate more time to patient care—findings that are consistent with previous studies that emphasise AI's ability to streamline processes and enhance patient care (Hogg et al. 2023; Ayorinde et al. 2024). The potential for AI to improve pharmacological treatment could significantly reduce medication‐related errors, addressing a critical issue identified by the WHO. WHO reports that globally, one in 20 patients experiences preventable medication‐related harm, with over a quarter of these cases being severe or life‐threatening (WHO 2023). By enabling more accurate prescribing and monitoring, AI offers promising advancements in medication safety and efficacy.

The second synthesised finding identified AI impacts on professional roles and workforce dynamics that within healthcare. Healthcare professionals have expressed concerns about the impact of AI on workforce dynamics, including potential unemployment and a decline in professional competence. These concerns align with broader discussions about AI reshaping the global workforce (Chhibber et al. 2025). Perceptions related to time constraints and the need for training, as also identified by Rogan, Bucci, and Firth (2024), reflect the understanding that learning to use AI requires time and new types of competences, which directly influence job descriptions and role expectations. A lack of trust in AI systems and the demand for evidence further reflect apprehensions about professional change—how AI influences the role and responsibility of healthcare professionals, alters perceptions of autonomy and authority and raises questions about evolving relationships and task boundaries between healthcare professionals and AI.

The third synthesised finding highlights the importance of preserving human connection in patient care, even as AI becomes more integrated into healthcare practices. This reflects concerns raised in previous studies about AI's lack of empathy (Rogan, Bucci, and Firth 2024). While AI excels in technical support, it cannot replace the empathy and understanding that are pivotal to interactions between patients and healthcare professionals, as recognised previously in several studies (Miller and Mutha 2025). Future research should explore strategies for balancing technological advancements with maintaining the human touch in healthcare settings.

In the fourth synthesised finding, healthcare professionals identified various challenges associated with the implementation of AI, such as technical issues, information overload and financial challenges. These challenges align with the study by Lambert et al. (2023), which highlights that AI acceptance is closely tied to organisational factors such as legal liability and the availability of supporting infrastructure. Policymakers and healthcare organisations should collaborate to develop supportive frameworks that facilitate the smooth integration of AI while minimising disruptions to healthcare professionals' daily practices.

The fifth synthesised finding revealed healthcare professionals' concerns about the ethical use of AI, highlighting issues such as data privacy, cultural sensitivity and the need for clear regulatory frameworks to ensure responsible integration into healthcare. These ethical and cultural dilemmas underscore the importance of ethical considerations in AI development and implementation, and the need is also recognised in previous studies (Lambert et al. 2023; Murphy et al. 2021; Karimian et al. 2022). Together, these concerns underscore the need for a multidisciplinary approach to AI implementation, ensuring ethically respectful and equitable healthcare services for everyone.

Based on these synthesised findings, this review highlights broader considerations for the successful implementation and integration of AI in healthcare. First, healthcare professionals experience AI as both an enabler and a potential threat. At the same time that AI supports efficiency and clinical decision‐making, it may also challenge professional identity and the perceived value of their human expertise. Second, the responsibility for implementing AI cannot rely solely on individuals. Healthcare organisations must provide sufficient support, including training, resources and leadership, to support AI adoption. Third, as AI plays an increasing role in clinical decisions, there is a need to clarify the boundaries of responsibility between healthcare professionals and AI. These broader implications indicate that successful AI integration requires a strategic and holistic approach that acknowledges the transformative impact of AI on healthcare.

### 5.1 Clinical Implications

The results of this review provide a foundation for an individualised approach in designing and implementing training or educational interventions to assist healthcare professionals from diverse professions in adopting AI in clinical practice. Moreover, the insights from the review can guide healthcare organisations and operational managers to reconsider professional roles and responsibilities in different positions in relation to the expectations and guidelines of AI use in patient care as well as strengthening organisational practices to provide necessary supporting infrastructures and upskill healthcare professionals accordingly.

### 5.2 Limitations

While the study was conducted carefully and with methodological rigour, some limitations should be acknowledged. Firstly, although the search strategies were extensive, the search results may still be limited because of the broad and variable nature of AI definitions. Many technologies, such as robotic systems, may incorporate AI components but not always be clearly identified or referred to as AI in the studies. This could have led to relevant data being overlooked during the data screening process. Additionally, despite searching several databases, some relevant studies may not have been captured. The reference lists of the included studies were not screened, which limits the comprehensibility of the findings. Language limitations may also exclude relevant studies from the review. However, the studies were from diverse geographical regions, that is, low‐, middle‐ and high‐income countries. In addition, data extraction was conducted by one reviewer, yet the other members of the research group confirmed the extraction.

The decision to exclude physicians from the inclusion criteria after full text review must be acknowledged as a major limitation, as it narrowed the professional perspectives represented in the findings extensively. However, the decision was made to enable more focused and coherent synthesis and accentuate the voice of these less prominent professions, such as radiographers or pharmacists. Including physicians would have significantly broadened the volume of data, potentially compromising the clarity and manageability of the synthesis. In the future, it is valuable to synthesise specifically medical professionals' perceptions of AI in healthcare, as they are a central group in clinical decision‐making and AI implementation. Overall, this study synthesised findings from ten distinct healthcare professions, offering a wide‐ranging view of the field and enhancing the overall comprehensiveness of this review.

## 6 Conclusion

This systematic review provides insights into healthcare professionals' perceptions of AI in healthcare. While AI holds significant potential to enhance efficiency and improve patient outcomes, it is essential to address the concerns raised by healthcare professionals regarding workforce dynamics, communication and ethical considerations. These findings can inform and support the implementation of AI in healthcare. Furthermore, as the use of AI in healthcare has the potential to reshape the work tasks and competence requirements of healthcare professionals, the findings of this study may also be used to develop AI‐related education and training that meets the demands of future healthcare work.

## Funding

This work was supported by of the University of Oulu and The Academy of Finland PROFI 7 (352788).

## Conflicts of Interest

The authors declare no conflicts of interest.

## Data Availability

Data sharing not applicable to this article as no datasets were generated or analysed during the current study.
